# The deubiquitinase activity of CYLD is required for B cell differentiation

**DOI:** 10.1038/s41419-026-08555-x

**Published:** 2026-04-08

**Authors:** Athanasios Pseftogas, Jessica Bordini, George Gavriilidis, Michela Frenquelli, Alessandro Campanella, Alessandra Rovida, Gaia Morello, Marina Gerousi, Eleni Theodosiou, Styliani-Christina Fragkouli, Vasileios Vasileiou, Theodoros Sklaviadis, Dimitra Dafou, George Mosialos, Claudio Tripodo, Fotis Psomopoulos, Thomas H. Winkler, Kostas Stamatopoulos, Paolo Ghia, Konstantinos Xanthopoulos

**Affiliations:** 1 Vita-Salute San Raffaele University, Milan, Italy; 2 Division of Experimental Oncology, B cell neoplasia unit, IRCCS Ospedale San Raffaele, Milan, Italy; 3 Institute of Applied Biosciences, Centre for Research and Technology, Thessaloniki, Greece; 4 Tumor Immunology unit, University of Palermo, Palermo, Italy; 5 Department of Biology, National and Kapodistrian University of Athens, Athens, Greece; 6 Department of Molecular Biology and Genetics, Democritus University of Thrace, Alexandroupolis, Greece; 7 School of Pharmacy, Aristotle University of Thessaloniki, Thessaloniki, Greece; 8 School of Biology, Aristotle University of Thessaloniki, Thessaloniki, Greece; 9 Division of Genetics, Department of Biology, Friedrich-Alexander-Universität, Erlangen, Germany

**Keywords:** B-2 cells, NF-kappaB

## Abstract

CYLD is a functional deubiquitinase, involved in the regulation of significant cellular functions, including survival and apoptosis. To elucidate the role of CYLD in B cell differentiation, we generated transgenic animals with targeted deletion of the catalytically active form of the protein in B cells, starting from early differentiation stages. Our results indicate that catalytic inactivation of CYLD leads to a severe reduction of mature B cells, associated with blockade of differentiation at the Pro B cell stage, altered distribution of B cell populations in the spleen and bone marrow, culminating in impaired immune responses to model antigens. Single cell RNA sequencing of bone marrow B cells confirmed the severe perturbation of lymphopoiesis. Mechanistically, we found impaired expression of the IL-7 receptor alpha chain (IL-7Ra) and its upstream transcriptional activator FOXO1, leading to defective IL-7 signaling that is vital for early B cell development. However, the substrate(s) deubiquitinated by CYLD that regulates the FOXO1-IL-7R pathway remains unclear. Overall, our data imply a crucial role for the deubiquitinase activity of CYLD in B cell lymphopoiesis.

## Introduction

CYLD, encoded by the *Cyld* gene, is a deubiquitinase that removes linear (M1-linked) or K63-linked ubiquitin chains [1]. CYLD was discovered through its association with familial cylindromatosis, a benign skin tumor, wherein the appearance of the tumors was causally associated with mutations in the catalytic domain of the protein [2]. Subsequently, CYLD downregulation or inactivation has been associated with diverse solid [3–12] and hematological tumors [13–17]. CYLD is a negative regulator of multiple signaling events, including, nuclear factor-κB (NF-кB) [18–20], Wnt/β-catenin [21], and Notch signaling [22].

Although many splice variants of *Cyld* have been described, the most common encodes for a 956 amino acid long polypeptide. The catalytic domain of CYLD resides at its carboxy-terminal region, while three CAP-Gly domains are located in the amino-terminal region. CYLD also harbors a zinc-binding B-box domain and a BCL-3 binding domain in its central region. The first two CAP-Gly domains can interact with the carboxy-terminal tails of tubulin and microtubules [23], while the third interacts with the NF-κB essential modulator (NEMO). Between the second and the third CAP-Gly domains, two proline-rich motifs, a TRAF-binding motif and a phosphorylation region, have also been identified [24]. The complexity of the structure and the function of the molecule are further highlighted by the observation that constitutive expression of a carboxy-terminal truncated, thus catalytically inactive, form of the protein in mice is neonatally lethal [25, 26], while CYLD-knockout (*Cyld*^-/-^) mice, where the whole protein is not expressed, are viable [6, 27, 28].

CYLD has been implicated in diverse functions, including cell proliferation and control of cell cycle, cell apoptosis, inflammation, spermatogenesis as well as antiviral and immune responses [29]. While the role of CYLD in T cell development is well-defined [28], less is known about its involvement in B cell lymphopoiesis. Initial studies with *Cyld*^-/-^ mice showed lymphoid organ hyperplasia associated with increased spontaneous and induced activation of B cells, suggesting constitutive activation of NF-κB [30]. In contrast, other studies suggested a much lower impact of CYLD ablation on B cell maturation, with normal numbers of B cells in the spleen of two independently created *Cyld*^*-/-*^ mice [6, 27]. Interestingly, transgenic animals expressing a shorter CYLD splice variant (*Cyld*^*ex7/8*^) [31, 32], that retains its catalytic activity but lacks the central regulatory domain, exhibited larger spleens, lymph nodes and Peyer’s patches compared to controls.

The contrasting effects of CYLD knock-out in B cell lymphopoiesis insofar reported, may potentially derive from differences in the animal models used and the non-B cell restricted ablation of the deubiquitinating activity of the protein. Thus, we aimed at better elucidating the role played by CYLD in B cell lymphopoiesis by generating transgenic animals with B cell-specific catalytic inactivation of CYLD at early stages of B cell maturation, through the disruption of the catalytic-domain-coding region of the gene. By combining immunophenotyping and functional assays with next-generation sequencing, we here show that CYLD plays a major role in the maturation and function of B cells and that disruption of its catalytic activity severely impedes B cell responses.

## Materials and Methods

### Mouse models

Mb1Cre-Cyld^flx/flx^ mice were generated by crossing a neo^-^ derivative (*Cyld*^*flx/flx*^ *neo*^*-*^) of the previously developed *Cyld*^*flx/flx*^ mice [25] with *Mb1Cre* mice [33] (provided by Prof. M. Reth) [34]. In *Cyld*^*flx/flx*^ mice, loxP sites flank exon 9 and when recombination occurs in the presence of Cre recombinase, the ensuing exons, including the gene region encoding for the catalytic domain, are out of frame and not expressed. All mice were maintained under specific pathogen-free (SPF) conditions at the animal laboratory facility of the IRCCS Ospedale San Raffaele, Milan, Italy. Mice of experimental groups were age-matched (3 and 6 months), of both genders and, in most cases, littermates. Mice were genotyped (please refer to the following section) and assigned to the experimental groups based on their age and sex, maintaining in most cases a balance between the two sexes, without further randomization. All animal experimentation (including power analysis to estimate sample size) has been approved by the Animal Ethics Committee of the IRCCS Ospedale San Raffaele for compliance to European regulations and licensed by the Italian Ministry of Health (protocol numbers: 0018831-17/07/2015, 0021741-P-07/08/2019).

### Genotyping PCR

Genotyping of the mice was performed by polymerase chain reaction (PCR) analysis of genomic DNA. The following PCR primers were used to characterize the *Cyld* locus: Fn: 5′-GGATCACTGTTGCCATCCTT-3′, and Rn4: 5′-AAAAAGACCCCCAGCCTTTA-3′ to amplify the WT (250 bp) and floxed (300 bp) allele; FWD1: 5′-GATGGCTCTTGTCACCACTT-3′, F6: 5’-CGTGAACAGATGTGATGAAGG-3’ and R6: 5’-CTACCATCCCTGCTAACCA C-3’ to amplify the KO (250 bp) and floxed (400 and 1193 bp) allele. The presence of the *Cre* transgene was assessed by PCR of genomic DNA using the following primers: mb1wt_fwd: 5′-CTGCGGGTAGAAGGGGGTC-3′ and mb1wt_rev: 5′-CCTTGCGAGGTCAGG GAGCC-3′ to amplify the WT (400 bp) allele; mb1cre_fwd: 5′-CCCTGTGGATGCCACCTC-3′, and mb1cre_rev: 5′-GTCCTGGCATCTGTCAGAG-3′ to amplify the *Cre* (430 bp) transgene.

### Flow cytometry

Bone marrow (BM, from the femur and tibia) and spleen were harvested from each mouse following sacrifice by cervical dislocation under isoflurane anesthesia. Single cell suspensions were prepared in Phosphate Buffered Saline (PBS, Euroclone, ECB4053) and subjected to erythrocyte lysis, using ACK lysis buffer (154.4 mM ammonium chloride, 10 mM potassium bicarbonate, and 97.3 μM EDTA tetrasodium salt). Cells were incubated in PBS for 10 min at room temperature with fluorescently labeled antibodies (outlined in Table S1). IgM staining was indirect, using a biotinylated primary antibody and PE-conjugated streptavidin for detection. The markers used to identify specific cellular subpopulations are summarized in Table S2. Flow cytometry was performed using either a Gallios (Beckman Coulter) or a Cytek Northern Lights (Cytek Biosciences) flow cytometer, while the readings were analyzed using FCS Express Cytometry v7 software (Denovo Software) or SpectroFlo software (Cytek Biosciences).

### Cell survival

Single-cell suspensions were prepared from bone marrow and spleen. Bone marrow and spleen samples were subjected to erythrocyte lysis using ACK lysis. The total and live cell numbers were counted using Trypan Blue (Sigma-Aldrich, T8154-EA) exclusion with a TC20 Automated Cell Counter (Bio-Rad).

### Immunoblotting

Cells were rinsed twice with ice-cold PBS and lysed with SDS lysis buffer (50 mM Tris-HCl pH 6.8, 2% w/v SDS, 10% v/v glycerol, and 3% v/v β-mercaptoethanol), followed by heating at 95 °C for 5 min. The samples were analyzed by SDS-PAGE, and proteins were electrophoretically transferred to a nitrocellulose membrane for Western blot analysis. Non-specific binding to the membrane was prevented using a 5% w/v BSA (Sigma-Aldrich, A9418) solution in PBST 0.1% (Phosphate Buffered Saline with Tween20 0.1% v/v) for 1 h at room temperature. Immunoblotting was performed using the antibodies presented in Table S3, diluted in blocking buffer. Membrane-bound antibodies were detected by an enhanced chemiluminescence detection kit (Bio-Rad, 1705061) using a ChemiDoc imaging system (Bio-rad, 17001401).

### Histopathology and immunohistochemistry

Spleen, bone marrow, and Peyer’s patches from the wall of the small intestine were collected and fixed in 10% neutral buffered formalin overnight, washed in water, and paraffin-embedded.

Four-micrometer-thick sections of mouse tissue were deparaffinized, rehydrated and unmasked using Novocastra Epitope Retrieval Solutions pH 6 (RE7113) and pH 9 (RE7116-CE) in a water bath at 98 °C for 30 min. Subsequently, the sections were brought to room temperature and washed in PBS. After neutralization of the endogenous peroxidase with 3% v/v H_2_O_2_ and Fc blocking by 0.4% w/v casein in PBS (Novocastra), the sections were incubated with antibodies. The following primary antibodies have been used for immunohistochemistry (IHC) on murine tissue: CD3 (clone CD3-12, 1:100 pH9, Abcam), PAX5 (clone EPR3730(2), 1:1000 pH9, Abcam), Ki67 (1:1000 pH6, Abcam), B220/CD45R (clone RA3-6B2, 1:10 pH9, BD), CD21 (clone SP186, 1:100 pH9, Abcam), BCL6 (clone 7D1, 1:100 pH9, Abcam).

IHC staining was developed using the Novolink Polymer Detection Systems (Novocastra, RE7150-K) or IgG (H&L)-specific secondary antibodies (Life Technologies, 1:500) and DAB (3,3′-Diaminobenzidine, Novocastra, GK346810) as substrate chromogen. Double IHC staining was performed with the ImmPRESS™ anti-Rat Polymer Detection Kit (Vector Laboratories, MP-5444-NB) alkaline phosphatase-conjugated produced in goat and Vulcan Fast Red as substrate chromogen.

Photomicrographs were collected using a Zeiss Axiocam 503 color digital camera with Zen 2.0 software (Zeiss). Digitization of slides was performed using an Aperio CS2 digital scanner (Leica Biosystems) with ImageScope software. Photomicrographs were evaluated in a blind manner.

### Immunization and ELISA

Age-matched mice were immunized intraperitoneally once on day 0 with 100 μg of NP-CGG (Santa Cruz Biotechnology, Ratio 10-19, sc-396208) or 50 μg TNP-AECM-FICOLL (Santa Cruz Biotechnology, sc-281696). Sera were collected at the indicated time points. In order to detect antigen-specific antibodies, plates were coated with 5 μg/ml NP-CGG or TNP-AECM-FICOLL. Serum was added at a dilution of 1:5000 (v/v) in PBS and plates were incubated overnight at 4 °C. Bound serum antibodies were detected using SBA Clonotyping System-C57BL/6-AP kit (Southern Biotech, 5300-04B) according to the manufacturer’s instructions. The same kit was used to detect different types of immunoglobulins in naïve mice 3 days before immunization, by coating the plates with 5 μg/ml UNLB capture antibody (part of Southern Biotech, 5300-04B).

### Stimulation with TLR ligands

Single cell suspensions were prepared from spleen and samples were subjected to erythrocyte lysis using ACK lysis buffer. Isolation of CD19^+^ splenic B cells was achieved by negative selection (Stemcell Technologies, 19854) and isolated subpopulations were reanalyzed for purity by flow cytometry (purity 95%). 5×10^4^ isolated splenic B cells were placed on a round-bottom 96-well plate using RPMI 1640 medium containing 4.5 g/L of glucose (Euroclone, ECB2000L), 10% fetal bovine serum (Euroclone, ECS5000L), 100 U/µl of penicillin-streptomycin (Gibco-Invitrogen, 15140-122), 1 mM sodium pyruvate (Sigma, S8636), 10 mM Hepes (Gibco-Invitrogen, 15630080) and 50 mM β-mercaptoethanol (Gibco-Invitrogen, 31350010). Then, they were stimulated with 1 μg/ml LPS-EK (Invivogen, tlrl-eklps) or 2.5 μΜ CpG ODN1826 (Invivogen, tlr1826) for up to 72 h. The total and live cell numbers were counted using Trypan Blue (Sigma-Aldrich, T8154-EA) exclusion and the TC20 Automated Cell Counter (Bio-rad).

### Treatment with IL-7

Single cell suspensions from the BM were prepared as previously described and subjected to erythrocyte lysis with ACK lysis buffer. In vitro differentiation of murine B cells was performed as described in [34]. Briefly, 75 × 10^4^ BM cells were subjected to two rounds of culture (4 days each) in medium containing recombinant mouse IL-7 (R&D Systems, 407-ML) at a final concentration of 10 ng/ml. Between sequential cultures, cells were washed and re-plated at the initial concentration. After each round of culture, CD19^+^/B220^+^ cells were assessed by flow cytometry as previously described.

### Cell sorting and isolation

B cell subpopulations were sorted using a MoFlo Atrios cell sorter (Beckman Coulter). Briefly, single cell suspensions were prepared from the bone marrow (collected from the femurs) or the spleen and marked as follows: BM B cells: CD19^+^B220^+^; BM Pro-B cells: CD19^+^B220^+^c-kit^+^CD25^-^; bone marrow Pre-B cells: CD19^+^B220^+^c-kit^-^CD25^+^; spleen follicular zone B cells: CD19^+^ CD23^high^CD21^low^; spleen marginal zone progenitor B cells: CD19^+^CD23^int^CD21^int^; spleen marginal zone B cells: CD19^+^CD23^-/low^CD21^high^. Isolation of CD19^+^ BM and splenic B cells was achieved by negative selection (Stemcell Technologies, 19854). Both cell-sorted and isolated subpopulations were reanalyzed for purity by flow cytometry (purity 95%).

### Single-cell RNA sequencing

For single-cell RNA sequencing, sorted bone marrow CD19^+^B220^+^ B cells were collected in RPMI medium (Euroclone, ECB2000) supplemented with 10% FBS (Euroclone, ECS0180L). Cell viability was then determined using Trypan Blue exclusion (Sigma-Aldrich, T8154-EA) and TC20 Automated Cell Counter (Bio-Rad). 4000 cells were encapsulated using the Chromium Controller platform (10X Genomics) and libraries were prepared using the Chromium Next GEM Single Cell 3’ Kit v3.1 (10X Genomics). The final libraries were sequenced according to the manufacturer’s instructions on a Novaseq 6000 (Illumina) platform.

### Computational analysis of scRNAseq data

Single cell RNA sequencing yielded about 100 million paired-end reads per sample. Cellular barcodes corresponding to good-quality cells were identified and extracted using the UMItool pipeline, then reads were aligned to the mouse genome mm10, Gencode version M22. Selected cells were then analyzed using the workflow implemented in the R package Seurat [35] (v.4.0.6), in the R environment v.4.2.2. The data were filtered for the following parameters: gene present in >5 cells, cells with >200 genes, and percent of mitochondrial genes <10%. Cells expressing >6000 genes were excluded. Before main pre-processing, the Mb1Cre-Cyld^flx/flx^ dataset was downsampled to ~700 cells using the Leverage Score Sampling function of the “Atomic sketch integration for scRNA-seq data” pipeline [36] from Seurat (https://satijalab.org/seurat/articles/atomic_integration.html) to obtain comparable cell numbers from the two samples. Main pre-processing of the control Cyld^flx/flx^ and Mb1Cre-Cyld^flx/flx^ samples entailed normalization, scaling, regression of mitochondrial and cell-cycle genes using SCTransform v2 methodology [37], followed by PCA reduction on the first 30 principal components and then clustering and visualization through UMAP. Pre-processed Seurat objects were automatically annotated using the SingleR package [38] (Celldex:ImmGen database) and downstream analysis continued only with B cell populations. The B cell-specific Seurat objects were analyzed again (SCTransform v4, PCA on 30 principal components, clustering, UMAP) and low-granularity annotations were assigned accordingly (“Pro B”, “Pre B”, “Pro/Pre B”, “Immature B”, “Mature B”). Next, trajectory pseudotime analysis was conducted with the SCORPIUS package [39], using the embeddings from prior UMAP clustering of the two Seurat objects. Gene modules were retrieved based on the top-100 trajectory-related genes that constituted the most important features of the Random Forrest classifier powering the SCORPIUS tool. Lastly, the bespoke computational workflow we devised to in silico approximate signaling cascades post-ablation of Cyld’s catalytical activity (summarized in S8) dovetailed the scMINER package for single-cell Gene Regulatory Network (GRN) reconstruction using the SJARACNe algorithm and the Expression2Kinases (X2K) pipeline.

### Protein–protein interaction networks

Protein–protein interaction (PPI) networks were designed using STRINGdb v1 [40, 41], processed using Cytoscape (v.3.9.1) [42]. PPI networks have been thresholded by applying a cut-off of “STRING combined score” of 0.4 as edge weight.

### RNA extraction, cDNA synthesis, and quantitative Real-Time PCR

Total RNA extracted from sorted BM Pro- (CD19^+^B220^+^c-kit^+^CD25^-^) and Pre- (CD19^+^B220^+^c-kit^-^CD25^+^) B cells and used for bulk RNA sequencing, was also transcribed to cDNA using iScript™ cDNA Synthesis Kit (Bio-Rad, 1708890). Analysis of cDNA samples by Quantitative Real-Time PCR (RT-qPCR) was performed using the CFX Connect Real-Time PCR Detection System (Bio-Rad) and SsoAdvanced Universal SYBR Green Supermix (Bio-Rad, 1725271) according to the manufacturer’s instructions. The PCR program included 1 cycle at 95 °C for 10 min and 40 cycles at 95 °C for 15 s and at 60 °C for 1 min. The threshold cycle (CT) value for each gene was normalized to the CT value for 18S rRNA. Relative expression levels are depicted as 2^-ΔCt^ values. The sequences of primers used for qPCR are outlined in Table S4.

### Statistics

All datasets were taken from *n* ≥ 3 biological replicates, unless otherwise specified. Data are presented as mean ± SEM. Unless otherwise indicated, the calculation of *p* values was performed with two-tailed unpaired Student’s *t*-test using GraphPad Prism software (GraphPad Software, La Jolla California USA). A *p*-value < 0.05 was considered statistically significant.

## Results

### Generation of mice with targeted CYLD catalytic inactivation in B cells

To investigate the role of CYLD in B cell lymphopoiesis in vivo, we generated mice with specific CYLD inactivation in B cells. More specifically, mice that express the Cre recombinase gene under the control of *CD79a* promoter/enhancer elements (Mb1Cre mice [33]) were crossed with previously generated mice, in which loxP sites flanking *Cyld* exon 9 were introduced [25], thus eliminating the catalytic-domain-coding capacity of the gene (codons 507-932). The genetic background of the mice was C57BL/6.

The presence of the *Cyld*^*flx*^ (Fig. S1A) and the Mb1Cre allele (Fig. S1B) as well as the recombination of the *Cyld* gene (Fig. S1C) were confirmed by PCR in Mb1Cre-Cyld^flx/flx^ and Cyld^flx/flx^ mice. PCR results indicated that the *Cyld*^*flx/flx*^ transgene was expressed in all cells, however in Mb1Cre-Cyld^flx/flx^ mice, harboring the Cre recombinase gene under the control of the *CD79a* promoter, recombination occurred only in B cells (Fig. S1C). Cell lysates from splenic B cells isolated from either Mb1Cre-Cyld^flx/flx^ or Cyld^flx/flx^ mice were further analyzed by Western Blotting for the presence of CYLD, using an antibody that targets the carboxy-terminus of the protein, confirming lack of immunostaining in B cells derived from Mb1Cre-Cyld^flx/flx^ animals (Fig. S1D).

As the 9^th^ exon of the *Cyld* gene, encoding for amino acid residues 507–561 of the protein in the carboxy-terminus, was targeted through our genetic approach, we next assessed whether an amino-terminal fragment of the protein could still be expressed. To this end, we used primers targeting exons 2–3 or 11–12, upstream and downstream of the recombination site respectively, and observed that an mRNA corresponding to exons 2–3 of the gene was expressed in B cells extracted from both Cyld^flx/flx^ and Mb1Cre-Cyld^flx/flx^ mice, while mRNA corresponding to exons 11-12 was only transcribed in Cyld^flx/flx^ mice (Fig. S2).

### CYLD catalytic inactivation during early stages of B lymphopoiesis impairs bone marrow B cell development

**Fig. 1:**
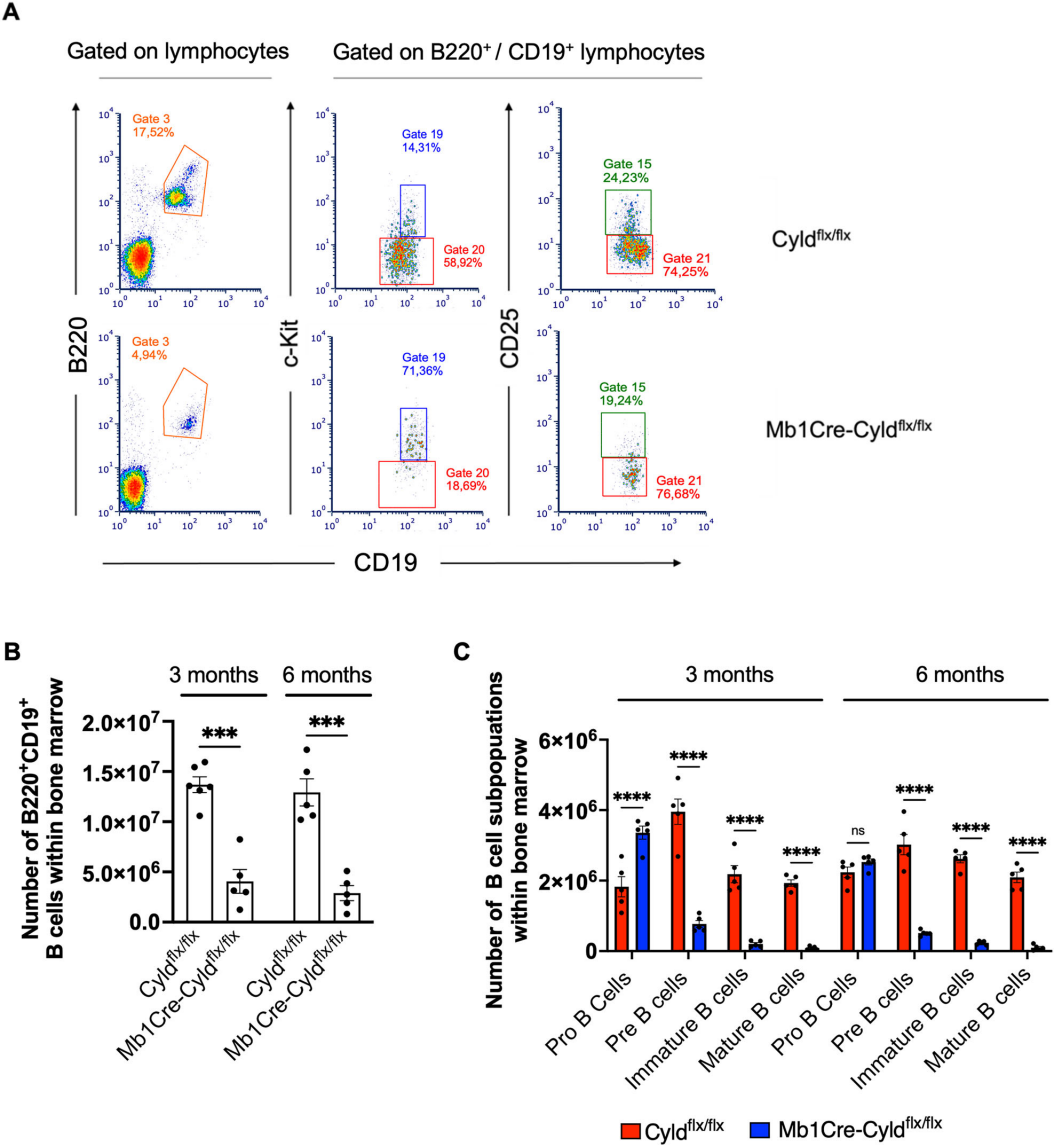
Immunophenotypic analyses of bone marrow B cells in Mb1Cre-Cyld^flx/flx^ mice. Cells from the bone marrow (femurs and tibia) of Mb1Cre-Cyld^flx/flx^ and Cyld^flx/flx^ mice at 3 and 6 months of age were prepared for immunophenotyping. Each point represents one individual, while lines show the mean ± SEM. **A** Representative staining of bone marrow B cells (CD19^+^ B220^+^), Pro-B cells (CD19^+^ B220^+^ c-kit^+^, Gate 19) and Pre-B cells (CD19^+^ B220^+^ CD25^+^, Gate 15) at 3 months of age. **B** Number of total B cells in Mb1Cre-Cyld^flx/flx^ and Cyld^flx/flx^ mice at 3 (*n* = 6 for Mb1Cre-Cyld^flx/fl^ and *n* = 5 for Cyld^flx/flx^) and 6 months of age (*n* = 5). **C** Number of the Pro-, Pre-, immature and mature B cell subpopulations within bone marrow in Mb1Cre-Cyld^flx/flx^ (*n* = 5) and Cyld^flx/flx^ (n = 5) mice at 3 and 6 months of age. Statistical differences were assessed by unpaired t-student analysis (**B**) and two-way ANOVA followed by Bonferroni’s multiple comparison test (**C**), comparing the mean between two groups (*** *p* < 0.001, **** *p* < 0.0001).

We initially assessed the potential impact of CYLD catalytic inactivation on early B cell development by analyzing cells from the BM of Mb1Cre-Cyld^flx/flx^ mice at 3 and 6 months of age. The total BM cellularity (Fig. S3A) and, in particular, the number of total B220^+^ CD19^+^ B cells were reduced in Mb1Cre-Cyld^flx/flx^ mice compared to control, Cyld^flx/flx^ mice (at 3 months: 1.47 ± 0.16×10^7^ vs 4.15 ± 0.94 ×10^6^, *p*-value < 0.001; at 6 months: 1.29 ± 0.13 ×10^7^ vs 2.9 ± 0.74 ×10^6^, *p*-value < 0.01) (Fig. 1A, B). Interestingly, Mb1Cre-Cyld^flx/flx^ mice displayed a higher amount of CD19^+^ckit^+^ Pro-B cells than control mice at 3 months (3.36 ± 0.18 ×10^6^ vs 1.82 ± 0.28 ×10^6^, *p*-value < 0.0001), while at 6 months, the number of Pro-B cells was comparable between the two groups (2.52 ± 0.08 ×10^6^ vs 2.23 ± 0.15 ×10^6^, p-value = 0.64) (Fig. 1A, C). Pro-B cell percentages were also higher at 6 months of age (Fig. S4B). Additionally, the number of CD19^+^CD25^+^ Pre-B cells was markedly reduced in Mb1Cre-Cyld^flx/flx^ compared to control Cyld^flx/flx^ mice both at the 3-month (3.95 ± 0.36 ×10^6^ vs 0.77 ± 0.09 ×10^6^, *p*-value < 0.0001) and the 6-month time point (3 ± 0.28 ×10^6^ vs 0.51 ± 0.03 ×10^6^, p-value < 0.0001) (Fig. 1A, C). Immature and mature B cell populations (Table S2) were also significantly down-represented in Mb1Cre-Cyld^flx/flx^ mice at both 3 (immature: 2.18 ± 0.24 ×10^6^ vs 0.20 ± 0.04 ×10^6^; mature: 1.93 ± 0.09 ×10^6^ vs 0.08 ± 0.02 ×10^6^) and 6 months (immature: 2.61 ± 0.11 ×10^6^ vs 0.23 ± 0.02 ×10^6^; mature: 2.1 ± 0.15 ×10^6^ vs 0.09 ± 0.03 ×10^6^, Fig. 1C, S3B) of age. We thus concluded that conditional disruption of CYLD starting from the Pro-B cell stage results in a severe impairment of B cell development, associated with accumulation of Pro-B cells and a significant reduction of the immature and mature B cell compartments in the bone marrow.

### CYLD catalytic inactivation affects splenic B cell distribution

**Fig. 2:**
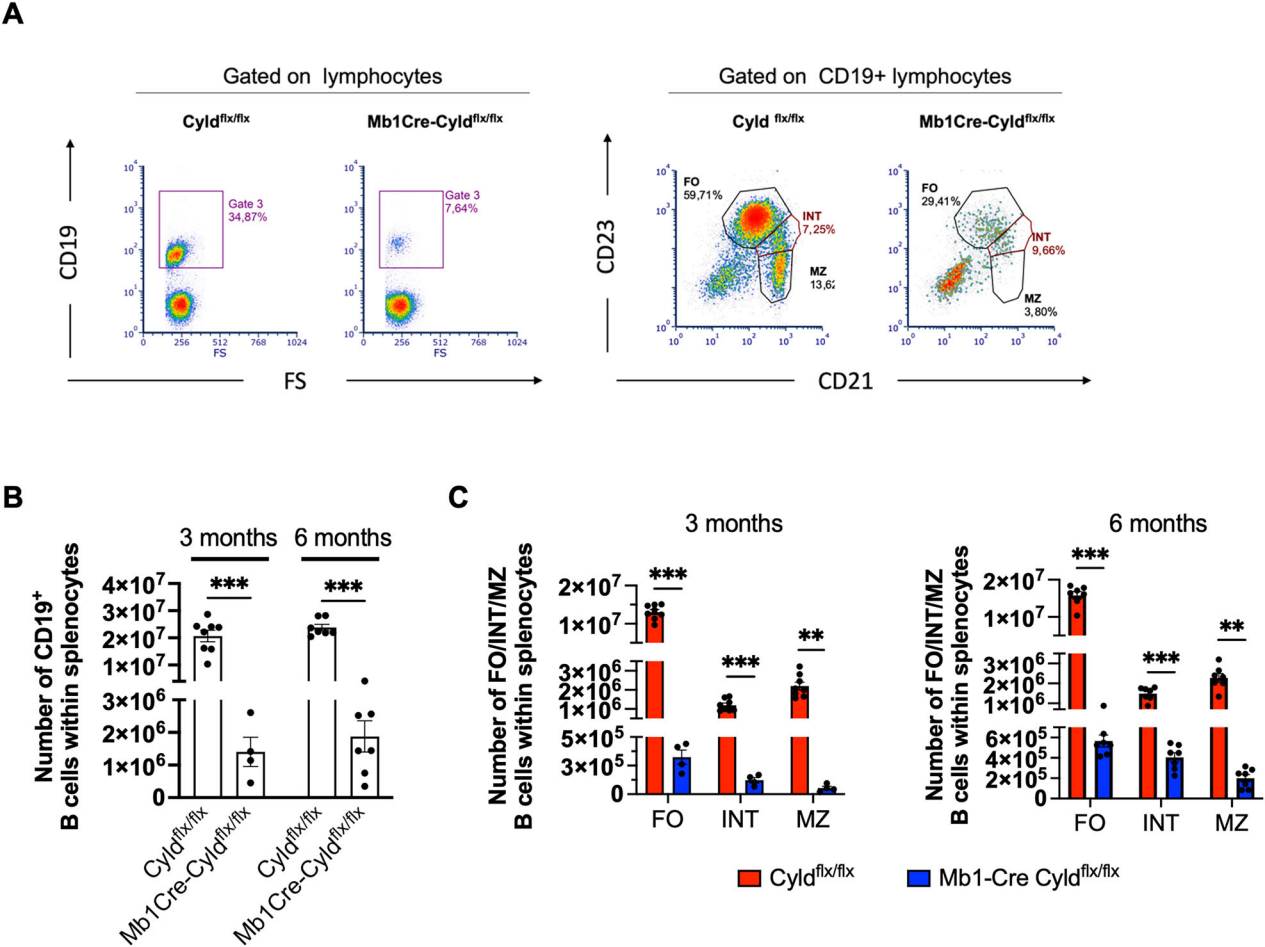
Immunophenotypic analyses of splenic B cells in Mb1Cre-Cyld^flx/flx^ mice. Splenic cells from Mb1Cre-Cyld^flx/flx^ and Cyld^flx/flx^ mice at 3 (*n* = 8 for Mb1Cre-Cyld^flx/flx^ and *n* = 4 for Cyld^flx/flx^) and 6 months of age (*n* = 7) were prepared for immunophenotyping. Each point represents one individual, while lines show the mean ± SEM. **A** representative staining of splenic B cells (CD19^+^) at 3 months of age (FS corresponds to forward scatter). Follicular zone B cells (FO, CD23^high^ CD21^int^), marginal zone B cells (MZ, CD23^low^ CD21^high^) as well as an intermediate population (INT, CD23^int^ CD21^int^) at 3 months of age were identified. **B** number of total B cells (CD19^+^) in Mb1Cre-Cyld^flx/flx^ and Cyld^flx/flx^ mice at 3 and 6 months of age. **C** number of the follicular zone, marginal zone and intermediate B cells subsets in Mb1Cre-Cyld^flx/flx^ and Cyld^flx/flx^ mice at 3 and 6 months of age. Statistical differences were assessed by unpaired t-student analysis comparing the mean between two groups (** *p* < 0.01, *** *p* < 0.001).

Having established that catalytic inactivation of CYLD affects early B cell development in the BM with consequences also on mature B lymphocytes, we next aimed at assessing its effects on spleen development. Macroscopical examination of spleen samples revealed a significant reduction in its size and weight in the Mb1Cre-Cyld^flx/flx^ mice at both 3 months (size: 10.9 ± 0.3 mm vs 13.1 ± 0.3 mm, *p*-value < 0.000001; weight: 57.0 ± 10.6 mg vs 81.7 ± 6.2 mg, p-value = 0.000180) and 6 months (size: 11.9 ± 0.1 mm vs 14.3 ± 0.3 mm, *p*-value < 0.000001; weight: 64.5 ± 6.3 mg vs 88.8 ± 6.2 mg, *p*-value ≤ 0.000001) of age (Fig. S5A–C). In agreement with this, the total cell count in the spleen was also significantly perturbed in Mb1Cre-Cyld^flx/flx^ mice (at 3 months: 1.9 ± 0.50 ×10^7^ cells vs 5.8 ± 0.57 ×10^7^ cells, *p*-value < 0.0001 and 6 months: 2.6 ± 0.71 ×10^7^ cells vs 6.7 ± 0.93 ×10^7^ cells, *p*-value < 0.0001), compared to Cyld^flx/flx^ mice (Fig. S5D). Immunophenotyping confirmed that the number of B cells was significantly reduced compared to control Cyld^flx/flx^ mice (Fig. 2A, B), while the residual B cells showed an altered distribution in terms of subpopulations. In detail, the relative amounts of transitional (T1; B220^+^IgM^high^IgD^low^ and T2; B220^+^IgM^int^IgD^int^) and mature (M; B220^+^IgM^low^IgD^high^) B cells were significantly reduced in Mb1Cre-Cyld^flx/flx^ mice compared to controls (Fig. S5E). Mb1Cre-Cyld^flx/flx^ mice at 3 months of age showed a marked reduction of both follicular (FO; CD23^high^ CD21^int^, 3.23 ± 0.62 ×10^5^ vs 1.30 ± 0.07 ×10^7^, *p* value < 0.0001) and marginal zone (MZ; CD23^low^ CD21^high^, 0.52 ± 0.16 ×10^5^ vs 2.19 ± 0.20 ×10^6^, *p*-value < 0.01) B cells (Fig. 2C, left panel). Additionally, a subset of B cells with intermediate expression of the FO and MZ B cell markers (INT, CD23^int^ CD21^int^) was significantly decreased (1.46 ± 0.43 ×10^5^ vs 1.17 ± 0.12 ×10^6^, *p*-value < 0.001). Interestingly, while the distribution of FO, INT and MZ cells remained similar at 6 months of age, there was a marked expansion of INT cells in Mb1Cre-Cyld^flx/flx^ mice (from 1.46 ± 0.43 ×10^5^ to 4.05 ± 0.45 ×10^5^) (Fig. 2C, right panel). As expected, there was no difference observed in the distribution of T cells between Mb1Cre-Cyld^flx/flx^ and control mice (Fig. S6A, B). These results demonstrate that CYLD catalytic inactivation in the early stages of B cell development also impacts B cell maturation in the spleen, leading to significant alterations in both the architecture and the cell distribution between the follicular and the marginal zone.

### CYLD catalytic inactivation leads to reduced Pax5^+^ B cell populations in the bone marrow and spleen

**Fig. 3:**
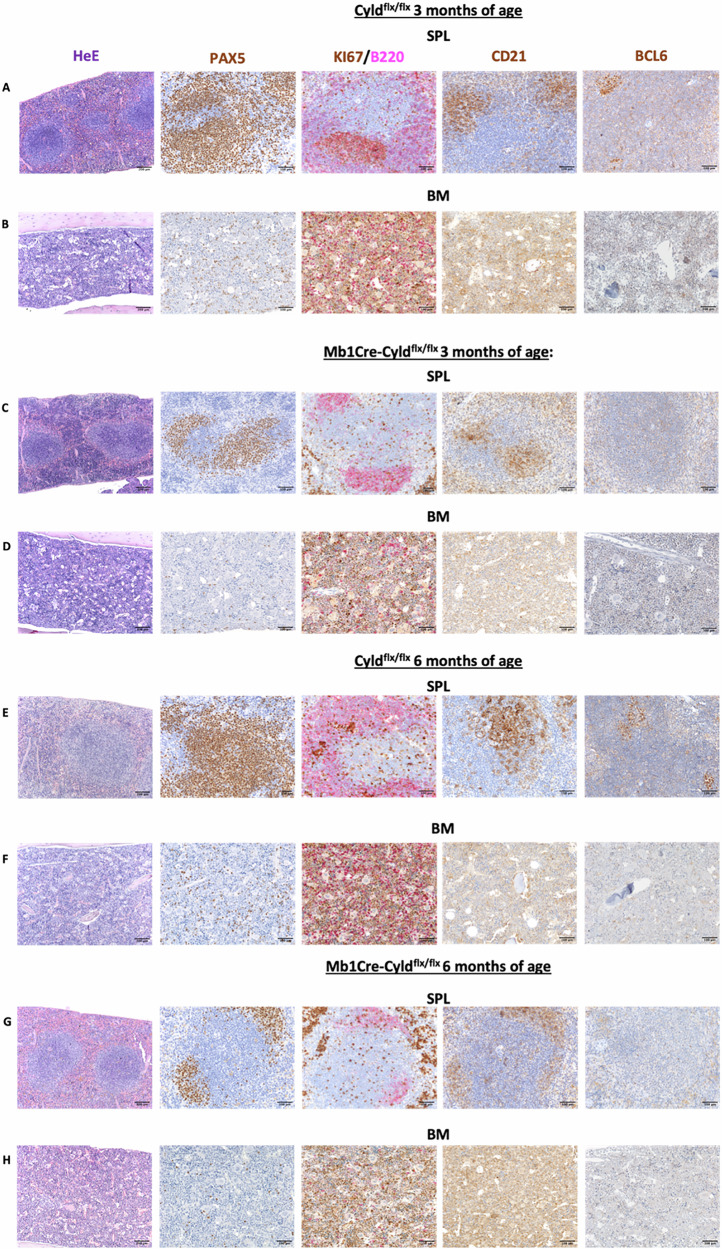
Immunohistopathological assessment of Mb1Cre-Cyld^flx/flx^ mice. Representative stainings of splenic (SPL, **A, C, E, G**) and bone marrow (BM, **B, D, F, H**) sections from Mb1Cre-Cyld^flx/flx^ and Cyld^flx/flx^ mice at 3 and 6 months of age (*n* = 4) with Hematoxylin/Eosin (H & E) at original magnification x100 (Scale bars, 250 μm), PAX5, KI67/B220, CD21 and BCL6 at original magnification x200 (Scale bars, 100 μm).

Histopathological analysis of spleen samples from 3-month-old Cyld^flx/flx^ mice showed normal overall composition of the spleen parenchyma with preserved white pulp architecture (PALS, B cell follicles and marginal zone) and red pulp populated mostly by erythroid elements and histiocytic myeloid cells with sparse megakaryocytes (Fig. 3A). IHC for the pan-B-cell marker Pax5 revealed normal numbers and distribution of these cell types (Fig. 3A). In addition, Cyld^flx/flx^ mice showed a normocellular hematopoietic BM with conserved myeloid-to-erythroid ratios and normal maturation of granulocytic and megakaryocytic elements (Fig. 3B).

Corresponding samples from 3-month-old Mb1Cre-Cyld^flx/flx^ mice exhibited an increase in the red pulp/white pulp ratio due to the slight decrease in the extent of Pax5^+^ B220^+^ CD21^+^ BCL6^-^ elements in the marginal zone and of follicular Pax5^+^ BCL6^+^ fractions (Fig. 3C). These changes became more prominent in samples from 6-month-old animals: the splenic white pulp of Mb1Cre-Cyld^flx/flx^ mice featured only residual foci of Pax5^+^ B220^+^ CD21^+^ BCl6^-^ B cells (Fig. 3G) due to a marked decrease of Pax5^+^ fractions.

The decrease in Pax5^+^ cells residing in the splenic white pulp of Mb1Cre-Cyld^flx/flx^ mice (Fig. 3C) was paralleled by a reduction in the frequency of Pax5^+^ cells in the BM that was mild at 3 months (Fig. 3D), becoming more evident at 6 months (Fig. 3H).

### CYLD catalytic inactivation affects splenic B cell responses to in vitro stimulation

To examine the activation and proliferation capacity of B cells in the absence of catalytically active CYLD, splenic B cells were purified by negative selection from Mb1Cre-Cyld^flx/flx^ and Cyld^flx/flx^ mice and cultured in vitro, in the presence or absence of TLR ligands (LPS or CpG ODN1826), used to induce cell activation and proliferation. Cell cultures were monitored daily for cell survival by cell counting.

In the absence of TLR stimulation, the number of B cells originating from Cyld^flx/flx^ mice remained rather stable over 72 h of culture (Fig. S7), while the cell population originating from Mb1Cre-Cyld^flx/flx^ mice doubled in the first 48 h and tripled at 72 h. When B cells from Cyld^flx/flx^ animals were stimulated with either LPS or CpG ODN1826, their number almost doubled every 24 h in the presence of LPS-EK or quadrupled when CpG ODN1826 was added. On the other hand, when LPS and CpG ODN1826 were added to cells from Mb1Cre-Cyld^flx/flx^ mice, the cell number did not increase compared to the cultures in the absence of TLR stimulants. These results indicate altered B cell responses to stimulation after B cell-specific CYLD inactivation.

### CYLD catalytic inactivation in early stages of B cell development impairs effective immune responses in vivo

**Fig. 4:**
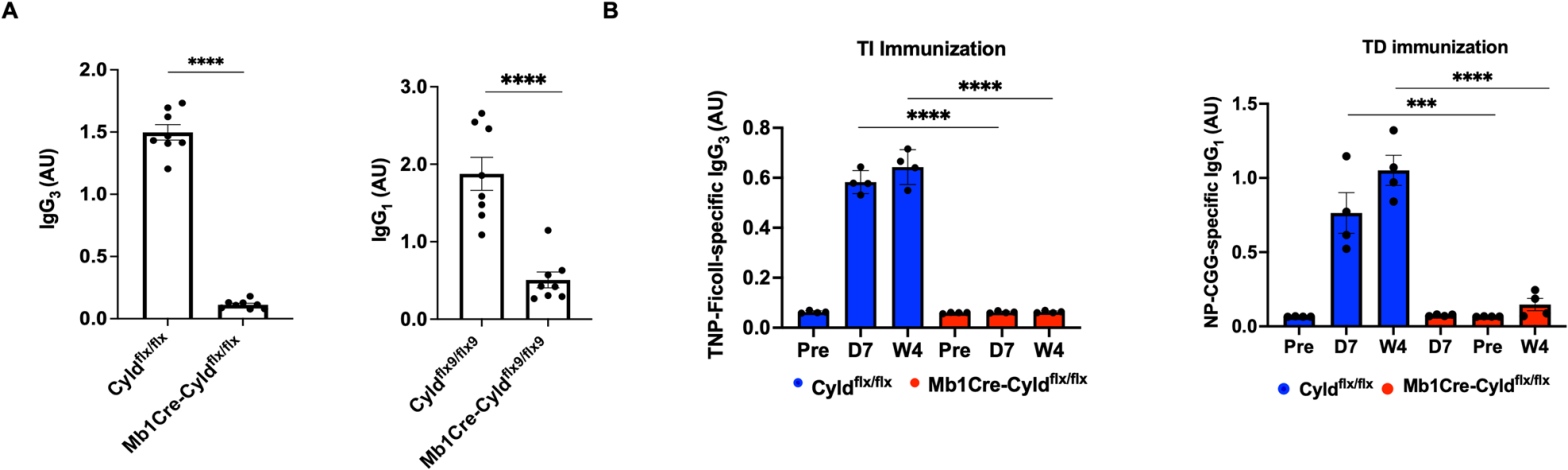
Mb1Cre-Cyld^flx/flx^ leads to perturbed immune responses. **A** IgG_3_ and IgG_1_ immunoglobulin levels of Mb1Cre-Cyld^flx/flx^ mice. IgG_3_ (Left panel) and IgG_1_ (Right panel) were analyzed in the sera of SPF-housed naïve Mb1Cre-Cyld^flx/flx^ and Cyld^flx/flx^ mice at 3 months of age. Pan-IgG_3_ and pan-IgG_1_ antibodies were used as a capture antibody in sandwich ELISA assays and IgG_3_ as well as IgG_1_ levels were analyzed in 8 Cyld^flx/flx^ and 8 Mb1Cre-Cyld^flx/flx^ mice. Each dot corresponds to one individual, lines represent mean ± SEM. Statistical differences were assessed by t-student analysis comparing the mean between two groups (**** *p* < 0.0001). **B** Immune responses of Mb1Cre-Cyld^flx/flx^ mice. T-cell independent immune responses (Left panel) were analyzed by sandwich ELISA in 4 Cyld^flx/flx^ and 4 Mb1Cre-Cyld^flx/flx^ mice immunized with TNP-Ficoll. Following immunization, sera were collected after 7 days and 4 weeks and TNP-Ficoll-specific IgG_3_ levels were determined. T-cell dependent immune responses (Right panel) were analyzed by immunizing 4 Cyld^flx/flx^ and 4 Mb1Cre-Cyld^flx/flx^ mice with NP-CGG. 7 days and 4 weeks after immunization, sera were collected and NP-CGG specific IgG_1_ levels were determined by sandwich ELISA. In all cases, IgG_3_ and IgG_1_ levels were analyzed as optical density in arbitrary units (AU). Each dot corresponds to one individual, lines represent mean ± SEM. Statistical differences were assessed by t-student analysis (left panel) and two-way ANOVA followed by Bonferroni’s multiple comparison test (right panel), comparing the mean between two groups (***p < 0.001, ****p < 0.0001).

To assess effects on humoral immune responses, we first quantified total IgG_3_ and IgG_1_ levels in the serum of naïve, 3-month-old Mb1Cre-Cyld^flx/flx^ mice housed in SPF conditions and found almost undetectable IgG_3_ and significantly low IgG_1_ titers compared to control Cyld^flx/flx^ mice (Fig. 4A). Furthermore, Mb1Cre-Cyld^flx/flx^ and Cyld^flx/flx^ mice were challenged with a T cell-independent (TNP-Ficoll) or a T cell-dependent antigen (NP-CGG). As expected, control mice exhibited high TNP-Ficoll IgG_3_ and NP-CGG IgG_1_ specific titers during the 4 weeks after immunization, while the corresponding IgG_3_ and IgG_1_ titers of Mb1Cre-Cyld^flx/flx^ mice remained undetectable (Fig. 4B). Interestingly, basal IgM levels as well as TNP-Ficoll-specific IgM were comparable between the Mb1Cre-Cyld^flx/flx^ and Cyld^flx/flx^ mice, while CYLD catalytic inactivation led to significantly lower IgM titers at the 7^th^ day after challenging the mice with NP-CGG (Fig. S8). Taken together, these results suggest that catalytic inactivation of CYLD severely affects immune responses: in the case of T cell-independent immunization, antigen-specific IgM is produced, but no IgG3 or IgG1, whereas antigen-specific production of IgM, IgG1 and IgG3 is significantly perturbed when mice are immunized with a T cell-dependent antigen.

### Single-cell transcriptomics corroborate perturbed maturation of B cells in the bone marrow due to CYLD catalytic inactivation

To better understand how CYLD influences early B cell lymphopoiesis, we FACS-sorted CD19^+^ B220^+^ B cells from BM aspirates of Cyld^flx/flx^ and Mb1Cre-Cyld^flx/flx^ mice and performed single-cell RNA sequencing (scRNA-seq). In the Cyld^flx/flx^ sample, 12,274 genes and 629 cells were recovered, and four clusters of physiological B cell subsets (i.e., Pro-B cells, Pre-B cells, Immature and re-circulating Mature B cells) were designated. *Cyld* transcripts were mostly detected in Immature and Mature B cells (Fig. S9D). Conversely, in the Mb1Cre-Cyld^flx/flx^ sample, 14,660 genes and 695 cells were recovered, showing aberrantly accumulated Pro- and Pre- B cells, as well as an intermediate Pro-/Pre- phenotype, accompanied by dwindling Immature and Mature B cells, hence mirroring our previous in vivo findings (Fig. 1B, C). Noticeably, *Cyld* transcripts were scattered across all B cell subpopulations but mostly accumulated in Mature B cells (Fig. S10D).

**Fig. 5:**
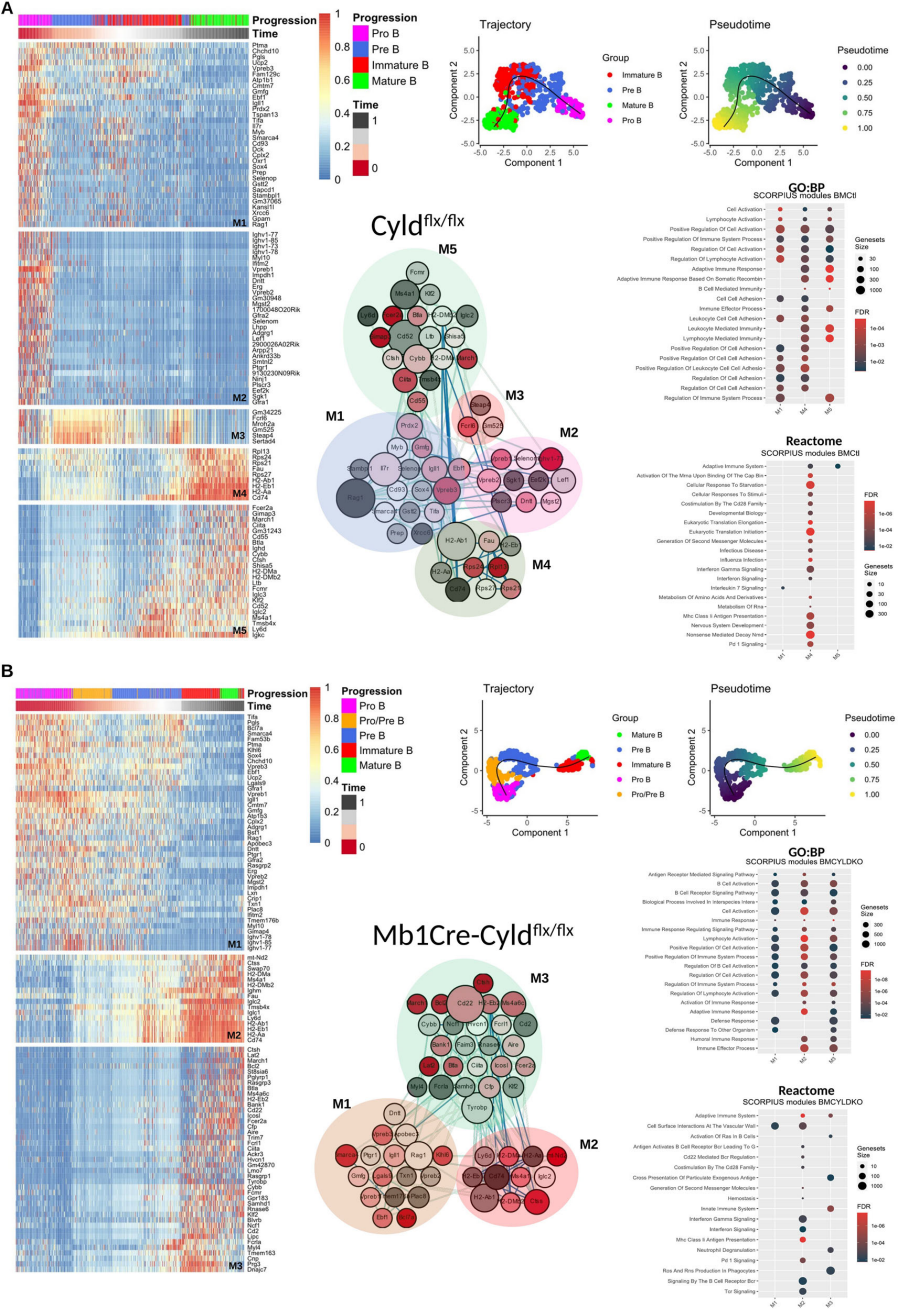
Mb1Cre-Cyld^flx/flx^ mice display a perturbed differentiation trajectory of maturing B cells, lacking Interleukin 7 regulation. Multi-panel figures depicting developmental stages of bone marrow B cells based on scRNA-seq trajectory pseudotime analysis for 1 control Cyld^flx/flx^ (**A**) and 1 Mb1Cre-Cyld^flx/flx^ sample (**B**). Each multi-panel figure depicts a heatmap showing gene modules across the trajectory pseudotime (left), UMAP clustering figures showing distinct B cell populations across the same trajectory pseudotime (upper right), a protein-protein interaction (PPI) network based on STRING database for important module genes (center) and pathway enrichment bubble plots for respective modules (center and lower right). In the PPI graph, the node color is analogous to the SCORPIUS module, the node size is analogous to the Betweenness Centrality of each gene while the width of the edges is proportional to the STRING combined score. Visualization of the graph was rendered in Cytoscape.

In terms of trajectory pseudotime and network-pathway enrichment, in the Cyld^flx/flx^ sample (Fig. 5A) we observed a normal, linear differentiation process spanning from Pro-B cells to Mature B cells, relevant trajectory-related genes (e.g., mainly *Rag1, IL-7R, H2-Ab1, Cd52* with high Between Centrality on the STRINGdb graph) and physiologically enriched pathways (e.g., physiological B cell differentiation and homeostasis, including IL-7 signaling, Adaptive Immune Responses based on Somatic Recombination). On the contrary, in the Mb1Cre-Cyld^flx/flx^ sample (Fig. 5B), we detected a disheveled differentiation process with paradoxical trajectory-related genes (e.g., negative regulators of B cell signaling *Cd22* and *Tyrobp* with high Between Centrality on the STRINGdb graph) and aberrantly enriched pathways (e.g., inflammatory responses, hyper-activation of innate and adaptive immunity and oxidative stress).

### Single-cell computational modeling prioritizes Il7 signaling along with ground-truth Nf-kB/TGF-beta pathways during CYLD catalytic inactivation

To gain insight into the underlying phenomena, we exploited the multi-dimensional nature of our B cell scRNA-seq datasets to create a computational representation of potential signaling cascades being deregulated by the ablation of CYLD’s catalytic activity.

To this purpose, we created Gene Regulatory Networks (GRNs) based on the SJARACNe algorithm (scMINER framework) [43] and calculated the differential activity of several driver molecules between Mb1Cre-Cyld^flx/flx^ and Cyld^flx/flx^ samples. The statistically significant hyper-active and hypo-active drivers were then provided as input to the X2K pipeline (Expression2Kinases) [44], which predicts upstream regulatory networks (kinases, intermediate molecules, TFs) associated with user-inputted sets of genes (outlined in Fig. S11).

**Fig. 6:**
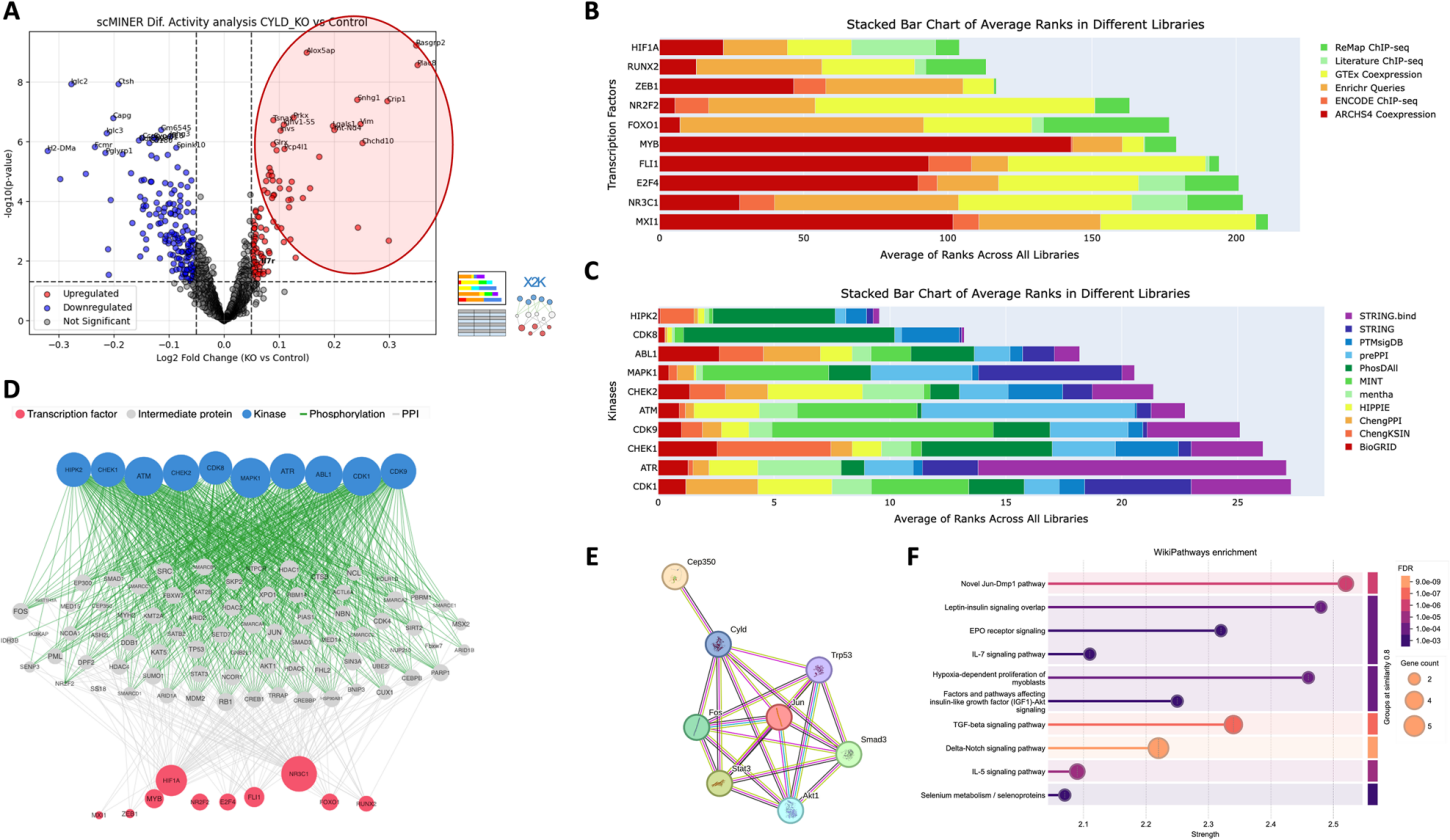
Computational scRNA-seq modeling on bone marrow B cells from the Mb1Cre-Cyld^flx/flx^ sample predicts unhinged IL-7 signaling due to the catalytic inactivation of CYLD. **A** Volcano plot emphasizing differentially hyper-active driver genes on the Mb1Cre-Cyld^flx/flx^ sample compared to the Cyld^flx/flx^ control sample, as provided from the scMINER GRN reconstruction tool. **B** Horizontally stacked barplots from the X2K workflow showing enriched transcription factors (TFs) for the top 50 hyper-active drivers from (**A**). **C** Horizontally stacked barplots from the X2K workflow showing enriched kinases for the top 50 hyper-active drivers from (**A**). **D** The X2K Kinases-Intermediates-TFs tripartite graph which computationally reconstructs major signaling cascades due to the catalytic inactivation of CYLD. **E** STRINGdb PPI network among CYLD and members of the X2K tripartite graph from (**D**). **F** Lollipop plot from STRINGdb depicting WikiPathways enrichment for the X2K tripartite graph from (**D**).

The first scMINER-X2K signaling model for hyper-active drivers identified several TFs (e.g.,*Hif1a, Runx2, ZEB1, Foxo1, Fli1*) and kinases (e.g., *Hipk2, Mapk1, Cdk1/8/9, Chek1/2*) that are well-documented modulators of B cell fate (Fig. 6A–C). By rendering the obtained tri-partite graph (Fig. 6D, Kinases-Intermediates-TFs) by this first scMINER-X2K model in the protein-protein interaction database STRINGdb along with the *Cyld* gene, we discovered (a) underlying connections of *Cyld* with several molecules like *Cep350, Trp53, Jun, Smad3, Akt1, Stat3, Fos* and (b) a significant enrichment of the cardinal CYLD-ground truth pathway TGF-beta signaling [45] along with Il-7 signaling pathway, Hypoxia, Selenium metabolism and Delta-Notch signaling which is a known signaling cascade of CYLD functionality (Fig. 6E, F). The cardinal implication of Il-7 pathway was in line with the previous scRNA-seq trajectory results and was further corroborated by the observation that the *Il7r* gene exhibited statistically significant hyper-activation in the Mb1Cre-Cyld^flx/flx^ sample (Supplementary file scMINER_DAG_results.csv).

**Fig. 7:**
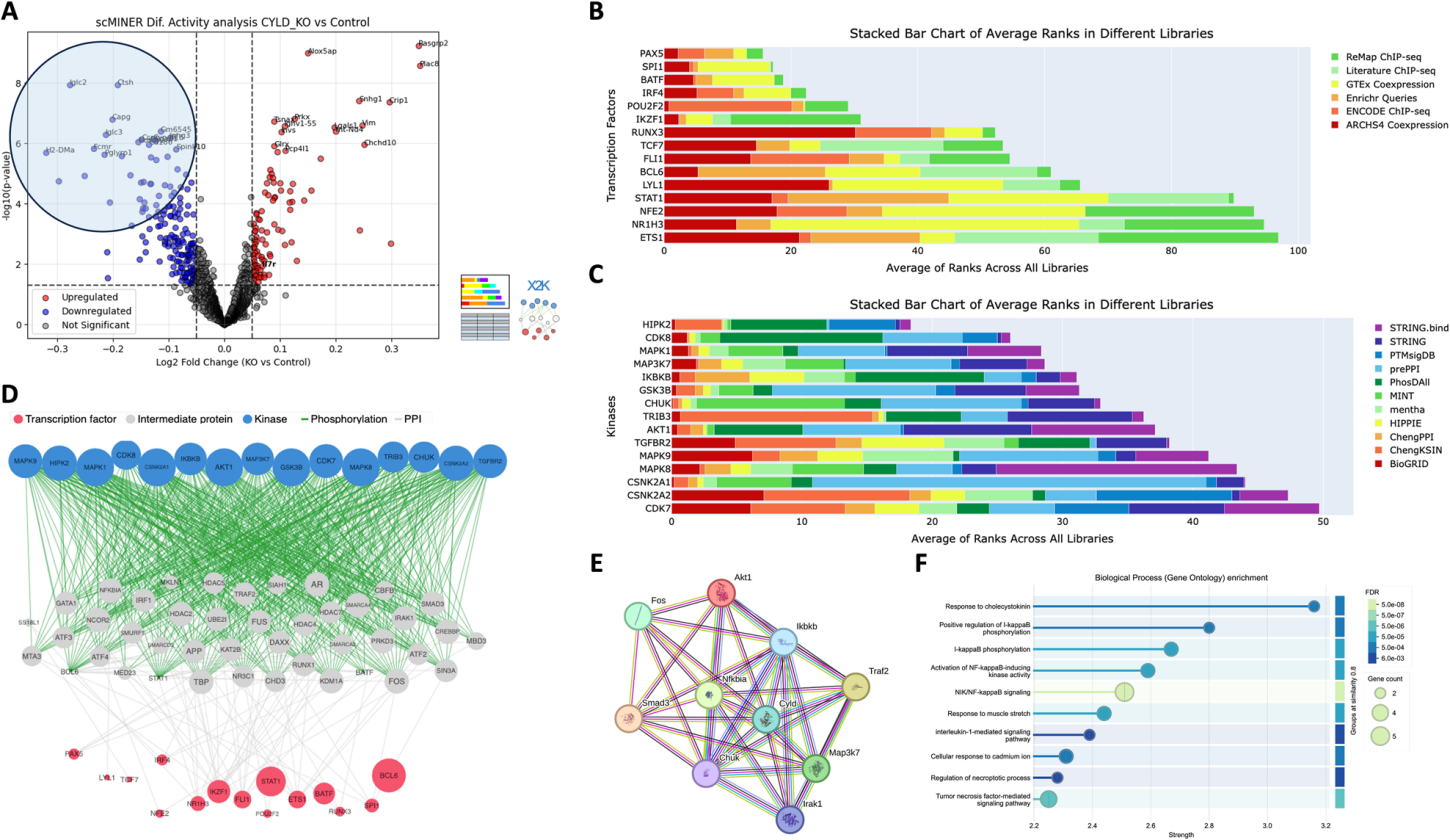
Computational scRNA-seq modeling on bone marrow B cells from the Mb1Cre-Cyld^flx/flx^ sample retrieves ground-truth Nf-kB biological pathway due to the catalytic inactivation of CYLD. **A** Volcano plot emphasizing differentially hypo-active driver genes on the Mb1Cre-Cyld^flx/flx^ sample compared to the Cyld^flx/flx^ control sample, as provided from the scMINER GRN reconstruction tool. **B** Horizontally stacked barplots from the X2K workflow showing enriched transcription factors (TFs) for the top 50 hyper-active drivers from (**A**). **C** Horizontally stacked barplots from the X2K workflow showing enriched kinases for the top 50 hyper-active drivers from (**A**). **D** The X2K Kinases-Intermediates-TFs tripartite graph, which computationally reconstructs major signaling cascades due to the catalytic inactivation of CYLD. **E** STRINGdb PPI network among CYLD and members of the X2K tripartite graph from (**D**). **F** Lollipop plot from STRINGdb depicting WikiPathways enrichment for the X2K tripartite graph from (**D**).

The second scMINER-X2K model based on the statistically significant hypo-active drivers also retrieved several TFs associated with B cell maturation (e.g*., Pax5, Irf4, Ikzf1, Runx3, Fli1, Ets1*) as well as B cell-related kinases (e.g., *Hipk2, Ikbkb, Gsk3b, Tgfbr2*, Fig. 7A–C), but the marked difference was that STRINGdb representation of the new tri-partite graph (Fig. 7D, Kinases-Intermediates-TFs) was predominantly associated with NF-kB pathway, which represents the most well-studied pathway of physiological CYLD functionality. In particular, *Cyld* exhibited connections with *Nfkbia, Ikbkb, Traf2, Map3k7, Irak1, Chuk, Smad3, Fos, and Akt1* (Fig. 7E). Interestingly, *Fli1*, a critical TF that participates in early and late B cell maturation [46, 47] and is associated with the NF-kB pathway (Supplementary File sc_MINER_DAG_results.csv), was identified as a key TF based on our analysis in both scMINER-X2K models and exhibited significant differential hypo-expression and hypo-activity in the Mb1Cre-Cyld^flx/flx^ sample.

To computationally evaluate the insofar findings more impartially, we constructed an expanded gene set based on mRNA co-expression patterns derived from the ARCHS4 database using *Cyld* as the seed gene in the Playbook Workflow Builder [48]. This independent in-silico data mining process successfully established a clear-cut connection of *Cyld* with *Il7r* and *Fli1* across the entire ARCHS4 database based on elevated Z-scores (Supplementary Table S5).

To validate our in-silico data obtained from both scMINER-X2K models, we sorted Pro-B and Pre-B cells from 3 Mb1Cre-Cyld^flx/flx^ and 3 Cyld^flx/flx^ mice, and we investigated the expression levels of key transcription factors involved in B cell maturation by qPCR. Genes encoding for transcription factors Ikzf1, Pax5, Ikzf3, Ebf1, Tcfe2a (E2A), Spif1 (PU.1), Flt3, Runx1, GFi1, Irf4, Irf8 and Miz-1 showed a significant upregulation in the Pre-B cell stage of Cyld^flx/flx^ mice, while the expression levels were considerably low in both Pro-B and Pre-B stages of Mb1Cre-Cyld^flx/flx^ mice (Fig. S12).

All together, these findings provide evidence for a perturbed regulation of critical transcription factors involved in B cell maturation, after CYLD catalytic inactivation, leading presumably to the block at the transition from the Pro-B to the Pre-B developmental stage.

### IL-7Ra expression and function are severely perturbed following CYLD catalytic inactivation

Since IL-7 signaling emerged from our scRNA-seq analysis as a cardinal signaling pattern disrupted by perturbation of CYLD catalytic activity, we queried bioinformatic databases for additional evidence on the potential interaction between perturbed CYLD expression and IL-7 signaling. We first used the L2S2 platform [49], which accesses the L1000 perturbation database and performed a query for potentially differentially expressed genes after CYLD KO experiments across available cancer cell lines. Through ensuing pathway enrichment via Enrichr (WikiPathways 2024 Mouse), we discovered that the connection between *CYLD* and IL-7 signaling was detected post *CYLD*-KO perturbations across the (a) differentially over- (*IRS1, STAT1, IRF1*) and under-expressed genes (*LYN, IL7R, MUC1*) in the HT29 colorectal cancer cell line and (b) in the differentially under-expressed genes (*BAX, CCND2, IRS1*) in the U251MG Glioblastoma Multiforme (GBM) cancer cell line (Supplementary Fig. S13). Moreover, *CYLD* and *IL7R* mRNA expression levels in lymphoid diseases were assessed by implementing the cBioPortal public database. Interestingly, we observed a significantly linear correlation of *CYLD* and *IL7R* mRNA expression levels in four independent datasets, representing mainly diffuse large B-cell lymphoma (Fig. S14). These results further corroborate the interplay between CYLD ablation and the coordinated signaling via the IL-7 signaling pathway.

**Fig. 8:**
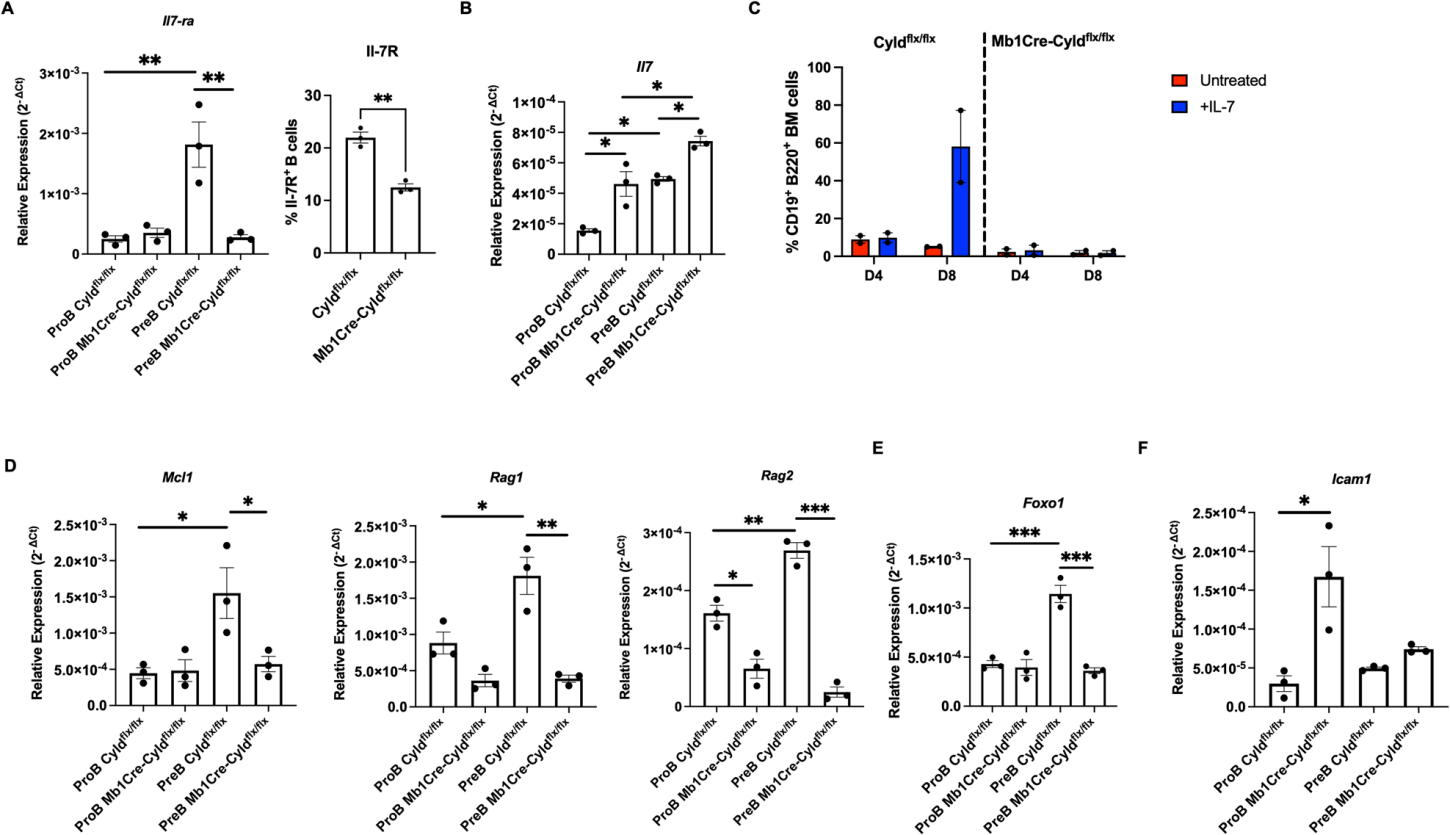
Catalytic inactivation of CYLD severely affects IL-7 signaling in Mb1Cre-Cyld^flx/flx^ mice. **A** Expression of IL-7Rα was determined at the gene level by qPCR in sorted Pro- (CD19^+^B220^+^c-kit^+^CD25^-^) and Pre- (CD19^+^B220^+^c-kit^-^CD25^+^) B cells from Mb1Cre-Cyld^flx/flx^ and Cyld^flx/flx^ mice (left panel, each point represents the mean value of technical triplicates from one individual, while lines show the mean ± SEM (*n* = 3)) and at the protein level by flow cytometry in bone marrow B cells (B220^+^ CD19^+^). The percentage of IL-7Rα^+^B cells in the bone marrow of Mb1Cre-Cyld^flx/flx^ and Cyld^flx/flx^ mice is graphed (*n* = 3). **B** Expression of interleukin 7 (Il7) was determined at the gene level by qPCR in sorted Pro- and Pre -B cells from Mb1Cre-Cyld^flx/flx^ and Cyld^flx/flx^ mice (*n* = 3). **C** Percentage of total bone marrow B cells (B220^+^ CD19^+^) was assessed by flow cytometry after 4-day and 8-day treatment with IL-7 (*n* = 2). **D** Mcl1, Bclxl, Rag1 and Rag2 expression was quantified at the gene level by qPCR in sorted Pro- and Pre-B cells from Mb1Cre-Cyld^flx/flx^ and Cyld^flx/flx^ mice (each point represents the mean value of technical triplicates from one individual, while lines show the mean ± SEM (*n* = 3)). **E** Relative expression of Foxo1 in sorted Pro and Pre- B cell from Mb1Cre-Cyld^flx/flx^ and Cyld^flx/flx^ mice determined by qPCR. Each point represents the mean value of technical triplicates from one individual, while lines show the mean ± SEM (*n* = 3). **F** Relative expression of Icam1 in sorted Pro and Pre- B cell from Mb1Cre-Cyld^flx/flx^ and Cyld^flx/flx^ mice determined by qPCR. Each point represents the mean value of technical triplicates from one individual, while lines show the mean ± SEM (*n* = 3). In (**A**), (**B**), (**D**), (**E**), and (**F**) statistical differences were assessed by t-student analysis (right panel, figure A) and two-way ANOVA followed by Bonferroni’s multiple comparison test, comparing the mean between two groups (* *p* < 0.05, ** *p* < 0.01, *** *p* < 0.001).

Based on these in silico findings, we next assessed the expression of *IL-7R*α, encoding the IL-7Rα subunit of the IL-7 receptor (IL-7R) in BM Pro-B and Pre-B cells, sorted from 3 Mb1Cre-Cyld^flx/flx^ and 3 Cyld^flx/flx^ mice. *IL-7Rα* expression levels were analyzed by qPCR and shown to be progressively upregulated in Cyld^flx/flx^ mice, from the Pro- to the Pre-B cell stage, as expected. In contrast, in Mb1Cre-Cyld^flx/flx^ mice, despite levels of expression similar to Cyld^flx/flx^ mice at the Pro-B cell stage, no upregulation of *IL-7Rα* expression was evident in the transition to the Pre-B cell stage (Fig. 8A left panel). Downregulation of IL-7R was also confirmed at the protein level by flow cytometry, with half of the total B (B220^+^ CD19^+^) cells expressing IL-7R in the BM of Mb1Cre-Cyld^flx/flx^ compared to Cyld^flx/flx^ mice (Fig. 8A right panel). Moreover, since IL-7 is able to suppress IL-7Rα transcription [50], we evaluated *Il7* expression levels. At the Pro-B cell stage of Mb1Cre-Cyld^flx/flx^ mice, *Il7* expression levels were found to be significantly increased and approaching the levels of the Cyld^flx/flx^ mice at the Pre-B cell stage (Fig. 8B), while *Il7* was further upregulated at the Pre-B cell stage of Mb1Cre-Cyld^flx/flx^ mice (Fig. 8B).

To assess potential functional consequences of IL-7Rα downregulation, bone marrow cells were cultured in the presence of IL-7 for 8 days and the total number of B cells (B220^+^CD19^+^) assessed by flow cytometry after 4 and 8 days of culture. Cells from Cyld^flx/flx^ mice responded to IL-7 stimulation, as evidenced by the increased percentage of total B cells, whereas cells from Mb1Cre-Cyld^flx/flx^ animals did not respond to the IL-7 stimulus, with their number remaining stable (Fig. 8C).

We next evaluated the expression of *Mcl1*, *Bclxl*, *Rag1* and *Rag2*, by qPCR as a read-out of active IL-7 signaling and observed that the expression patterns of all three genes mirrored IL-7Rα expression in both Pro- and Pre-B cells from Mb1Cre-Cyld^flx/flx^ and deviated considerably from Cyld^flx/flx^ mice, indicating that *IL-7Rα* downregulation in Pre B cells of Mb1Cre-Cyld^flx/flx^ mice also associates with an impairment of IL-7 signaling and related gene modifications downstream the pathway.(Fig. 8D).

Expression of *IL-7Rα* is regulated by Forkhead box transcription factor O1 (FOXO1) [51]. We thus assessed the expression levels of *Foxo1* by qPCR in Pro- and Pre-B cells sorted from Mb1Cre-Cyld^flx/flx^ and Cyld^flx/flx^ mice. In contrast to Cyld^flx/flx^ mice, where *Foxo1* is considerably upregulated during the transition from the Pro-B to the Pre-B state, in Mb1Cre-Cyld^flx/flx^ mice *Foxo1* expression remained low at both the Pro- and the Pre-B cell stage (Fig. 8E), likely contributing to the compromised expression of IL-7Rα.

Of note, we observed significantly increased NF-κB signaling pathway activation at the Pro-B cell stage of Mb1Cre-Cyld^flx/flx^ mice, as assessed by determining the expression levels of *Icam1*, a known target gene of NF-κB signaling pathway (Fig. 8F).

## Discussion

To better elucidate the role of CYLD in B cell lymphopoiesis, we generated a transgenic animal model with targeted catalytic inactivation of CYLD from the early stages of lymphopoiesis (Mb1Cre-Cyld^flx/flx^). In these animals, Pro-B cells accumulate in the bone marrow, whereas the subsequent differentiation stages are significantly perturbed, with severe reduction in immature, transitional and mature B cell compartments. Similar perturbations are also evident at later stages of peripheral maturation in the spleen, where both marginal zone (CD23^low^ CD21^high^) and follicular (CD23^high^ CD21^int^) B cells are markedly reduced, while a B cell population with intermediate immunophenotype (CD23^int^ CD21^int^) accumulates. These intermediate cells resemble CD21^high^ CD23^+^ splenocytes that are considered the immediate precursors of marginal zone B cells, deriving from T2 or the follicular B cell pool [52]. The observed changes in lymphopoiesis are also associated with changes in the size, cellularity and microarchitecture of the spleen. Moreover, bone marrow cells from animals with targeted catalytic inactivation of CYLD did not respond to stimulation with IL-7, while splenic B cells showed an intrinsic proliferative capacity in ex vivo cultures where, in contrast to control animals, they robustly proliferated without the need of TLR stimuli. Critically, catalytic inactivation of CYLD led to significant functional impairment, as Mb1Cre-Cyld^flx/flx^ mice failed to mount detectable immune (IgG_3_ and IgG_1_ mediated) responses when immunized with T-dependent or T-independent immunogens. Of note, immunization with a T-independent antigen generated an initial IgM response, even in animals with catalytic inactivation of CYLD, indicating a perturbation in class-switching.

scRNA seq in B cells from the BM of Mb1Cre-Cyld^flx/flx^ mice further confirmed at the molecular level that CYLD catalytic inactivation results in a significant perturbation of B cell lymphopoiesis, mirroring the immunophenotyping results, with a very early block in the differentiation process. scRNA seq analysis revealed the appearance of a Pre/Pro B cell cluster between the actual Pro- and Pre-B cell clusters. Whether this additional cluster might be present also in control mice, but is too short-lived or of too limited size to be detected, or these cells arise specifically in Mb1Cre-Cyld^flx/flx^ mice as a result of the defective differentiation signaling they receive, warrants further investigation at higher resolution. As a result, the differentiation trajectory is significantly altered, affecting also later differentiation stages in the spleen, and leading to severe immunodeficiency. Interestingly, analysis in differentiating B cells from control animals, indicated an upregulation of *Cyld* expression at the Pre-B cell stage, while its expression returned at basal levels in the subsequent differentiation steps. This temporally well-defined upregulation of *Cyld* expression could orchestrate the ensuing differentiation steps, by providing crucial signaling cues that are missing in Mb1Cre-Cyld^flx/flx^ animals.

Furthermore, based on our work on differentially active drivers in single-cell omics [53], using GRN reconstruction coupled with computational predictions of upstream cell signaling pathways (*TFs-intermediate messages-kinases*, scMINER-X2K pipeline), we corroborated the involvement of the IL-7 signaling pathway along with Nf-kB /TGF-beta pathways. One predicted TF that emerged from our scRNA-seq pipeline was the *Friend Leukemia integration 1 transcription factor* (*Fli1)*. Disruptions in the physiological function of Fli1 can lead to marked attenuation of follicular B cells, imbalances in MZ B cells and accumulation of immature precursor B cells [46, 47]. In addition to the similarities of these published results with our Mb1Cre-Cyld^flx/flx^ phenotype, it is interesting to note that we were able to establish an independent in-silico association between *Cyld*, *Il7r* and *Fli1* through co-expression analysis in the massive bulk RNA-seq-based ARCHS4 database; further in vitro experimentation should be pursued in the future to delineate the potential Fli-1-Cyld-Il7r interplay.

Our scRNA-seq pipeline also hinted at a perturbation of AKT signaling. It has recently been suggested that AKT activity is crucial for the development of marginal zone B cells both in mice and humans [54]. Since Mb1Cre-Cyld^flx/flx^ mice also present with significantly reduced marginal zone B cells, the perturbation of AKT signaling could be a mechanism leading to impaired marginal zone differentiation.

Analysis of scRNA seq data, corroborated by qPCR, flow cytometry, independent bioinformatic databases (cBioPortal, L2S2 – L1000) and ex vivo functional studies, hinted at suppression of IL-7 signaling in Mb1Cre-Cyld^flx/flx^ animals as a key modification following CYLD catalytic inactivation. The signaling interplay between IL-7R and the pre-BCR has a crucial role in regulating B cell differentiation, by coordinating proliferation, mediated through IL-7R, and differentiation, driven by the pre-BCR [55]. Mutation of IL-7R leads to a severe blockade in B cell lymphopoiesis at the Pro- and Pre-B cell stage, while earlier developmental stages remain unaltered. That said, a small number of mature B cells appear in the periphery, confirming that maturation is feasible even in the absence of functional IL-7R or IL-7, though to a limited extent [56–58]. Thus, the perturbation affecting IL-7R in our model may well explain the impaired B cell differentiation including the Pro-B/Pre-B cell blockade in differentiation. Moreover, IL-7 synergizes with transcription factors, such as E2A, EBF and Pax-5 to coordinate B-cell lineage commitment, while additionally controlling Foxo1 and Rag activation [58]. In agreement with that, we confirmed that bone marrow cells originating from Mb1Cre-Cyld^flx/flx^ mice failed to respond to IL-7 stimulation and that the expression of key genes associated with B-cell differentiation and controlled through IL-7R, including *Ebf1*, *Tcfe2a*, *Pax5*, *Mcl1*, *Rag1* and *Rag2*, were perturbed. Trying to elucidate the mechanism leading to lower expression of IL-7R, we found that in the absence of catalytically active CYLD, the expression of *Foxo1*, an upstream transcriptional activator of IL-7 receptor alpha chain (IL-7Rα), is impaired in Mb1Cre-Cyld^flx/flx^ mice. It would be plausible to attribute this effect to CYLD-mediated deubiquitination of specific substrates. However, the identity of these substrates regulating the FOXO1-IL-7R pathway remains unclear. Furthermore, IL-7Rα expression might be transcriptionally suppressed by IL-7 or other pro-survival cytokines in T cells. Consistent with that, we observed significantly increased IL-7 expression levels from the Pro-B cell stage in Mb1Cre-Cyld^flx/flx^ mice, suggesting the possible presence of a similar regulating mechanism for IL-7R in B cells. Of note, signaling through the IL-7R is believed to play a cardinal role in B cell lymphopoiesis mostly in mice [59], though recent evidence indicates that it might also be important in human B cell lymphopoiesis, where it was shown that IL-7 controls commitment, proliferation and expansion of early B-cell progenitors, by regulating the expression of *BACH2*, *EBF1* and *PAX5* [60]. Whether similar regulatory networks involving CYLD catalytic activity and IL-7 signaling are also active in humans should be further investigated.

## Supplementary information

Supplemental material

Supplementary information

## Data Availability

Single cell RNA-seq data are deposited at Zenodo.org (https://zenodo.org/records/10124303?token=eyJhbGciOiJIUzUxMiJ9.eyJpZCI6IjUyZmIyNWFjLTBlOWItNDNlMi05NWFjLWVkYTI2YTIzNWQ2MyIsImRhdGEiOnt9LCJyYW5kb20iOiIwMjQ0NTcwOTJlOTUwYzljNjAzNGYxMjNlNTVmOTQ2YyJ9.QCtDq1LN61nqsNZ94dTGcXXDN1icwO-_iDOO_1_oVv5VGuG3-rktO4SYqDo). All relevant scripts, code and information about the bioinformatic analysis and computational models generated can be found at https://github.com/BiodataAnalysisGroup/Cyld-regulation-of-bcell-maturation. All other data are available from the corresponding authors upon request. Values for all data points in graphs are reported in the Supporting Data Values file.
