# Supplementary material for: The deubiquitinase activity of CYLD is required for B cell differentiation
Source: Cell Death Dis. 2026 Apr 8;17(1):496. doi: 10.1038/s41419-026-08555-x (PMC13187451; doi:10.1038/s41419-026-08555-x)
Supplement: Supplementary file 1 — Supplemental material [file 41419_2026_8555_MOESM1_ESM.docx]

**Supplemental material to “The deubiquitinase activity of CYLD is required for B cell differentiation”**

Supplemental Material list:

Fig. S1. Catalytic inactivation of CYLD occurring at the early stages of B cell differentiation.

Fig. S2. Expression of a putative N-terminal CYLD fragment.

Fig S3. Additional immunophenotyping of bone marrow B cells.

Fig. S4. Percentage of BM B cells in Cyld^flx/flx^ and Mb1Cre-Cyld^flx/flx^ mice.

Fig. S5. Macroscopical characteristics and further immunophenotyping of spleens.

Fig. S6. Percentage of splenic T cells in Cyld^flx/flx^ and Mb1Cre-Cyld^flx/flx^ mice.

Fig. S7. Mb1Cre-Cyldflx/flx leads to perturbed B cell proliferation.

Fig. S8. Immune responses of Cyld^flx/flx^ and Mb1Cre-Cyld^flx/flx^ mice.

Fig. S9. Cyld^flx/flx^ sample analysis with Seurat and SingleR computational tools.

Fig. S10. Mb1Cre-Cyld^flx/flx^ sample analysis with Seurat and SingleR computational tools.

Fig. S11. Graphical representation of the bespoke computational pipeline used to unravel signalling cascades in the scRNA-seq data.

Fig. S12. Expression levels of key transcription factors involved in B cell maturation.

Fig. S13-15. Correlation of CYLD expression with IL-7 pathway (L2S2 platform)

Fig. S16. Correlation of CYLD expression with IL-7 pathway (cBioPortal)

Fig. S17. Full and uncropped western blot for figure S1D

Table S1. Antibodies & other reagents used for flow cytometry.

Table S2. Cellular subpopulations identified by specific surface marker expression.

Table S3. Antibodies used for immunoblotting.

Table S4. Primer sequences used for qPCR.

Table S5. Genes similar to CYLD according to the mRNA Co-Expression from the ARCHS4 similarity matrix.

**Figure S1**


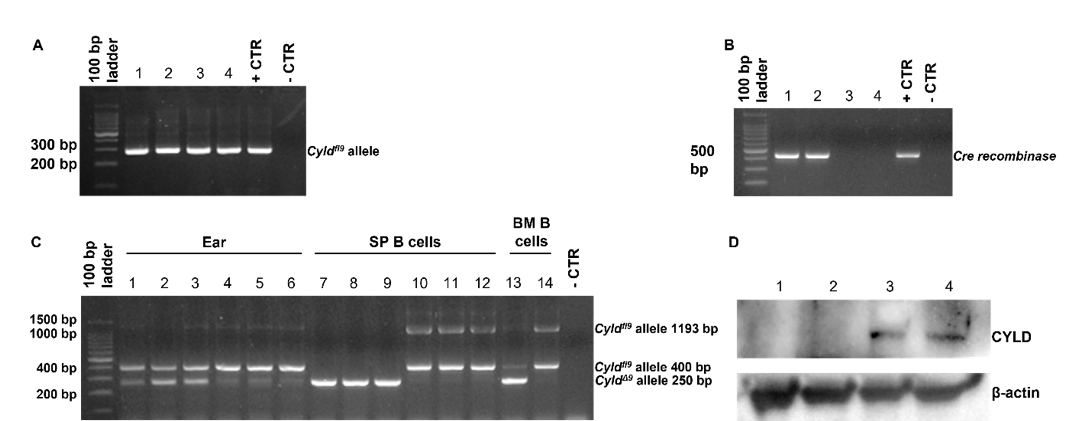


***Figure S1. Catalytic inactivation of CYLD occurring at the early stages of B cell differentiation.*** *PCR detection of the Cyld^flx^ allele* ***(A)*** *and the Mb1Cre allele* ***(B)*** *in genomic DNA from Mb1Cre-Cyld^flx/flx^ (lanes 1, 2) and Cyld^flx/flx^ (lanes 3, 4) mice.* ***(C)*** *PCR amplification of the Cyld exon 9 locus, in genomic DNA purified from the ear, splenic (SP) B cells or bone marrow (BM) B cells of Mb1Cre-Cyld^flx/flx^ (lanes 1-3, 7-9, 13) and Cyld^flx/flx^ (lanes 4-6, 10 -12, 14) mice. Following recombination, a shorter product is generated in splenic and bone marrow B cells from Mb1Cre-Cyld^flx/flx^ mice, but not in genomic material from Cyld^flx/flx^ mice. The shorter PCR product appears also in samples from the ears of Mb1Cre-Cyld^flx/flx^ mice. This is attributed to ectopic expression of Mb1Cre in chondrocytes.* ***(D)*** *CYLD detection by Western blotting using an anti-CYLD antibody in cell lysates from splenic B cells isolated from Cyld^flx/flx^ (lanes 1, 2) and Mb1Cre-Cyld^flx/flx^ (lanes 3, 4) mice.*

**Figure S2**

******

***Figure S2. Expression of a putative N-terminal CYLD fragment.*** *qPCR analysis of CYLD expression with primers targeting the first exons, upstream of the recombination site (Cyld^2-3^, left panel) and primers targeting exons downstream of the recombination site (Cyld^11-12^, left panel), using RNA isolated from sorted Pro- and Pre-B cells from Mb1Cre-Cyld^flx/flx^ or Cyld^flx/flx^ mice. Mb1Cre-Cyld^flx/flx^ mice express a truncated Cyld mRNA encoding for the N-terminal fragment of the protein. Cyld^flx/flx^ mice express the full length Cyld mRNA. Notice the upregulation in Cyld expression between the Pro and the PreB cell stage. Each point represents expression levels from one individual, while lines show the mean ± SEM. Statistical differences were assessed by two-way ANOVA followed by Bonferroni’s multiple comparison test, comparing the mean between two groups (* p<0.05).*

**Figure S3**

***Figure S3. Additional immunophenotyping of bone marrow B cells.*** ***(A)*** *Bone marrow cellularity.* ***(B)*** *Representative staining of bone marrow immature B cells (CD19^+^ B220^low^ IgM^int^) in Mb1Cre-Cyld^flx/flx^ mice. Each point represents one individual, while lines show the mean ± SEM. Statistical differences were assessed by t-student analysis comparing the mean between two groups (* p<0.05, **** p<0.0001).*

**Figure S4**

***Figure S4: Percentage of BM B cells in Cyld^flx/flx^* and *Mb1Cre-Cyld^flx/flx^ mice.*** ***(A)*** *Percentage of total B cells in Mb1Cre-Cyld^flx/flx^ and Cyld^flx/flx^ mice at 3 and 6 months of age.* ***(B)*** *Percentage of the Pro-B cell and Pre-B cell compartment in Mb1Cre-Cyld^flx/flx^ and Cyld^flx/flx^ mice at 3 and 6 months of age. Each bar represents mean and standard deviation from at least 3 animals per time point. Values are presented as the mean ± SEM. In* ***(A)*** *and* ***(B)*** *statistical differences were assessed by t-student analysis comparing the mean between two groups (* p<0.05, **** p<0.0001).*

**Figure S5**

***Figure S5. Macroscopical characteristics and further immunophenotyping of spleens.*** *Spleen size* ***(A, B)****, weight* ***(C)*** *and cellularity* ***(D)*** *in Mb1Cre-Cyld^flx/flx^ and Cyld^flx/flx^ mice.* ***(E)*** *Number of the T1, T2 and mature B cell compartment in Mb1Cre-Cyld^flx/flx^ and Cyld^flx/flx^ mice at 3 and 6 months of age. Each point represents one individual, while lines show the mean ± SEM. Statistical differences were assessed by t-student analysis comparing the mean between two groups (** p<0.01, *** p<0.001, ****p<0.0001).*

**Figure S6**

***Figure S6: Percentage of splenic T cells in Cyld^flx/flx^* and *Mb1Cre-Cyld^flx/flx^ mice.*** ***(A)*** *Percentage of total T cells in Mb1Cre-Cyld^flx/flx^ and Cyld^flx/flx^ mice at 3 and 6 months of age.* ***(B)*** *Percentage of the CD4^+^and CD8^+^ T cell compartment in Mb1Cre-Cyld^flx/flx^ and Cyld^flx/flx^ mice at 3 and 6 months of age. Each bar represents mean and standard deviation from 4 animals per time point. Values are presented as the mean ± SEM.*

**Figure S7**

******

***Figure S7. Mb1Cre-Cyld^flx/flx^ leads to perturbed B cell proliferation.*** *Splenic B cell proliferation ex vivo.* *B cells were isolated by negative selection from the spleens of 3 Mb1Cre-Cyld^flx/flx^ and 3 Cyld^flx/flx^ mice and cultured unstimulated or in the presence of LPS-EK (1 μg/ml) or CpG ODN1826 (2.5 μΜ). Each point represents one individual after 24h, 48h and 72h in culture, while lines show the mean ± SEM. Statistical differences were assessed by two-way ANOVA followed by Bonferroni’s multiple comparison test comparing the mean between two groups (***p<0.001, **** p<0.0001.*

**Figure S8**

******

***Figure S8. Immune responses of Cyld^flx/flx^ and Mb1Cre-Cyld^flx/flx^ mice. (A)*** *IgM was estimated in the sera of SPF-housed naïve Mb1Cre-Cyld^flx/flx^ and Cyld^flx/flx^ mice at 3 months of age. A pan-IgM antibody was used as a capture antibody in sandwich ELISAs and IgM levels were estimated in 8 Cyld^flx/flx^ and 8 Mb1Cre-Cyld^flx/flx^ mice.* ***(B)*** *T-cell independent immune responses were estimated by sandwich ELISA in 4 Cyld^flx/flx^ and 4 Mb1Cre-Cyld^flx/flx^ mice immunized with TNP-Ficoll. Following immunization, sera were collected after 7 days and 4 weeks and TNP-Ficoll-specific IgM levels were determined.* ***(C)*** *T-cell dependent immune responses were estimated by immunizing 4 Cyld^flx/flx^ and 4 Mb1Cre-Cyld^flx/flx^ mice with NP-CGG. 7 days and 4 weeks after immunization, sera were collected and NP-CGG specific IgM levels were determined by sandwich ELISA. In all cases, IgM levels were estimated as optical density in arbitrary units (AU). Each dot corresponds to one individual, lines represent mean ± SEM. Statistical differences were assessed by t-student analysis* ***(A)*** *and by two-way ANOVA followed by Bonferroni’s multiple comparison test* ***(B and C)****, comparing the mean between two groups (*p<0.05).*

**Figure S9**

*
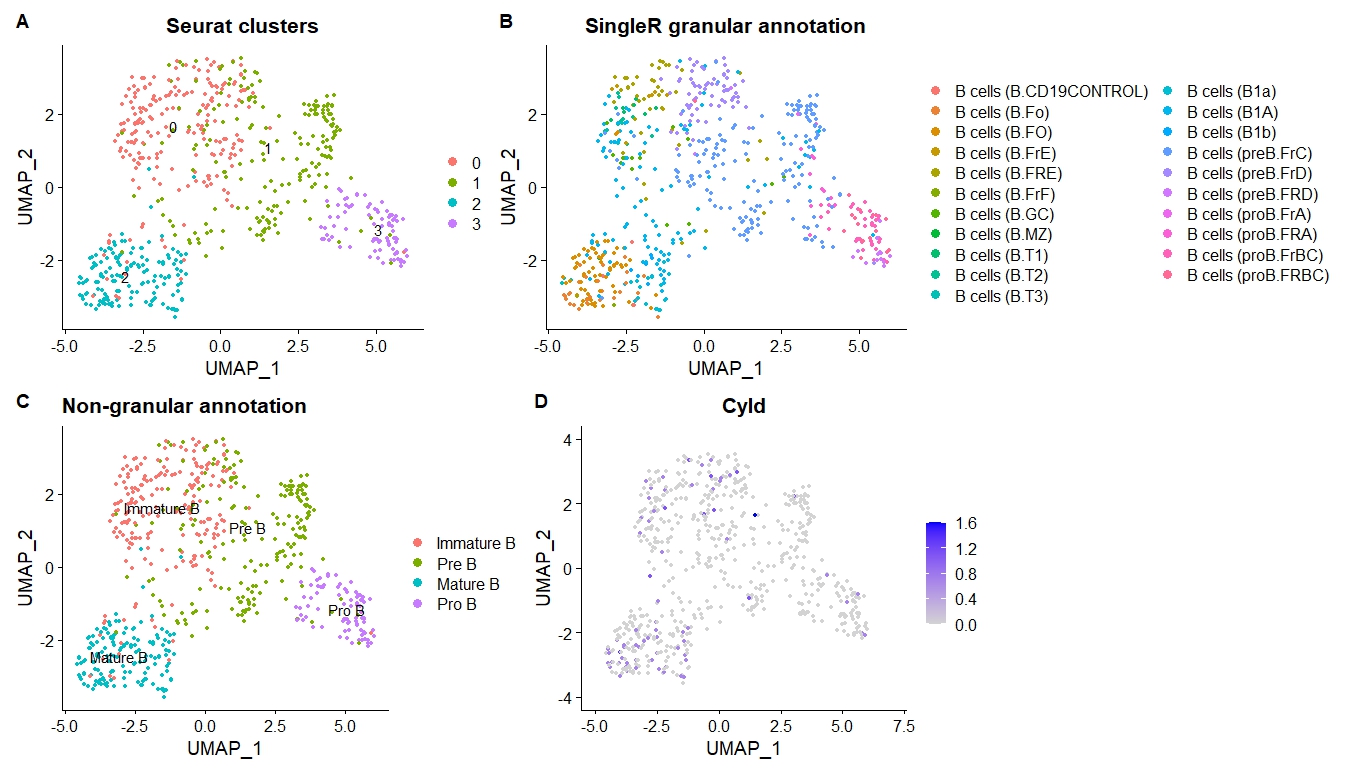
*

**Figure S9.** **Cyld^flx/flx^ sample analysis with Seurat and SingleR computational tools. (A)** UMAP clustering showing cell-groups in the Cyld^flx/flx^ sample based on Seurat pipeline. **(B)** UMAP clustering showing fine-grain annotation based on SingleR package (Celldex:ImmGen database). **(C)** Low-granularity annotation of Cyldflx^/flx^ sample for brevity. **(D)** UMAP clustering highlighting the expression of Cyld in the Cyld^flx/flx^ sample.

**Figure S10**


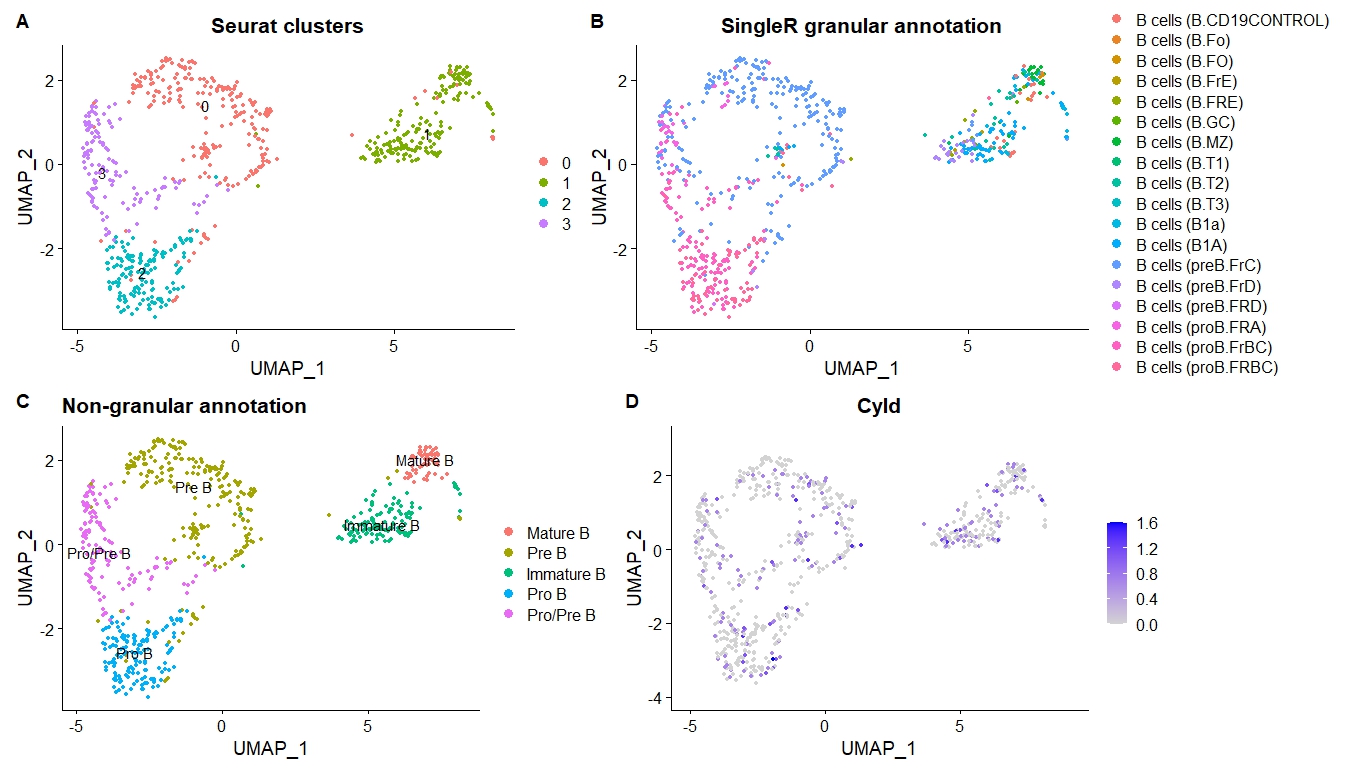


**Figure S10. Mb1Cre-Cyld^flx/flx^ sample analysis with Seurat and SingleR computational tools. (A)** UMAP clustering showing cell-groups in the Mb1Cre-Cyld^flx/flx^ sample based on Seurat pipeline. **(B)** UMAP clustering showing fine-grain annotation based on SingleR package (Celldex:ImmGen database). **(C)** Low-granularity annotation of Mb1Cre-Cyld^flx/flx^ sample for brevity. **(D)** UMAP clustering highlighting the expression of Cyld in the Mb1Cre-Cyld^flx/flx^ sample.

**Figure S11**

**
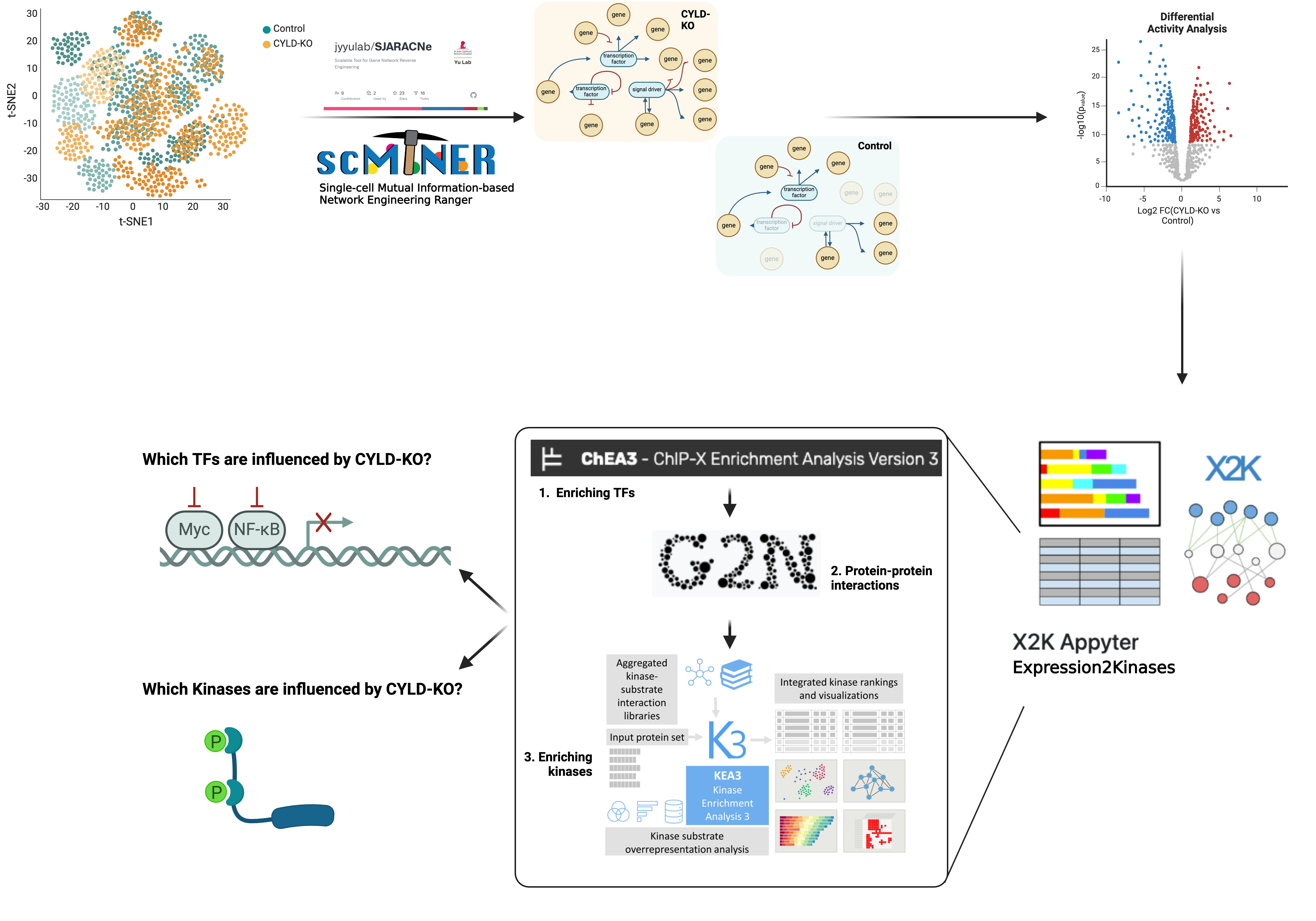
**

***Figure S11. Graphical representation of the bespoke computational pipeline used to unravel signalling cascades in the scRNA-seq data from Cyld^flx/flx^ sample the Mb1Cre-Cyld^flx/flx^ samples.*** *Initially, the scMINER tool ingests our scRNA-seq data to reverse-engineer Gene Regulatory Networks (GRNs) based on the SJARACNe algorithm and then calculates through driver molecules with statistically significant differential activity. Differentially hyper-active and hypo-active drivers are separately then provided as input to the X2K workflow; the latter identifies regulatory networks by linking user genes to transcription factors (via ChEA3), mapping their protein interactions, and then associating these with kinases using KEA3.*

**Figure S12**

***Figure S12. Expression levels of key transcription factors involved in B cell maturation.*** *The expression levels of Ikzf1, Pax5, Ikzf3, Ebf1, Tcfe2a (E2A), Spif1 (PU.1), Flt3, Runx1, GFi1, Irf4, Irf8 and Miz-1 were estimated by qPCR in RNA extracted from sorted Pro and Pre- B cells originating from 3 Mb1Cre-Cyld^flx/flx^ and 3 Cyld^flx/flx^ mice. Each point represents one individual, while columns show the mean ± SEM. Statistical differences were assessed by t-student analysis, comparing the mean between two groups (* p<0.05, ** p<0.01, *** p<0.001, **** p<0.0001).*

**Figure S13**


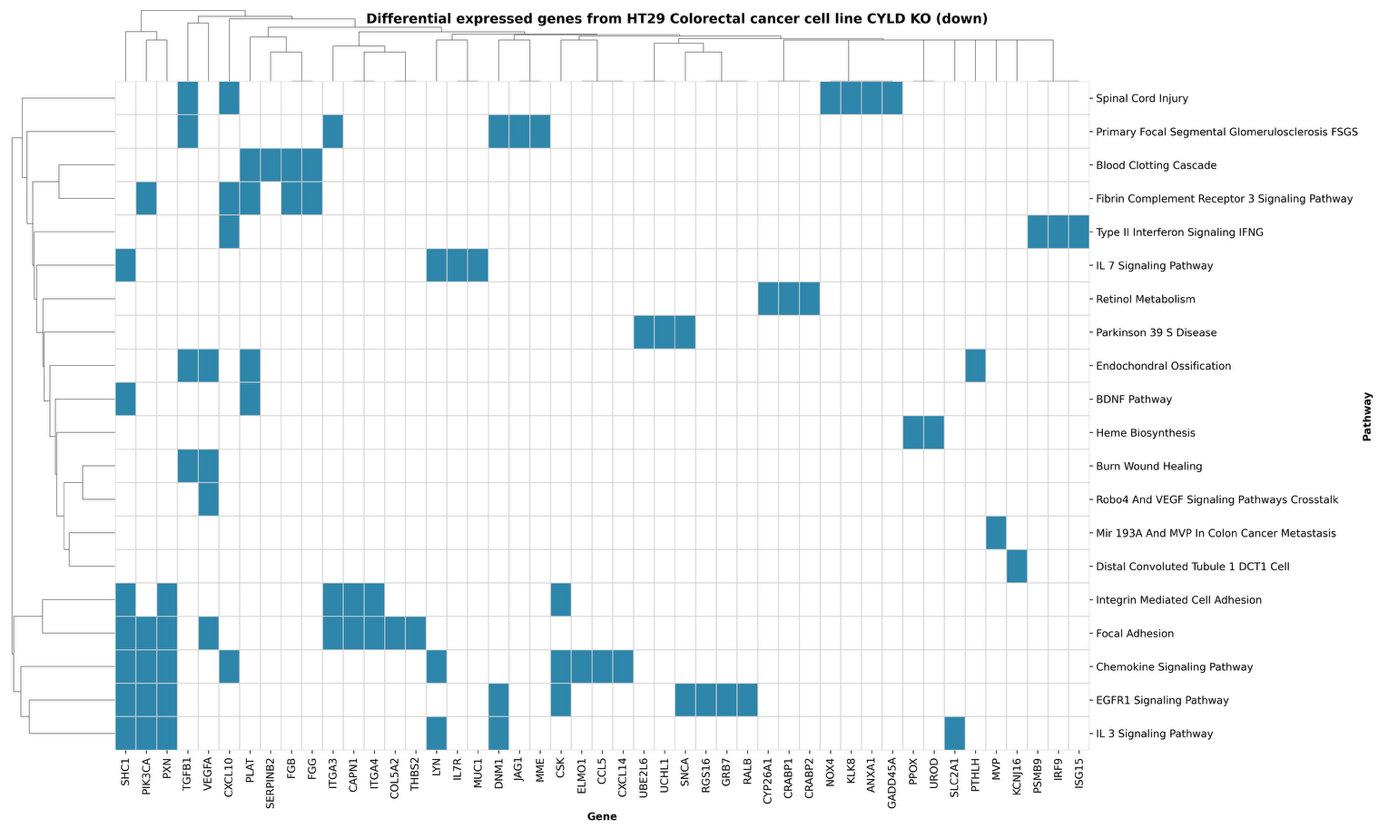


***Figure S13.*** ***Heatmap with hierarchical clustering depicting Enrichr pathway enrichment on differentially expressed genes (down) from the CYLD Knock-Out (KO) experiment on the HT29 Colorectal cancer cell line, as retrieved from the L2S2 web-tool.*** *The heatmaps show the top 20 pathways, thresholded by the Combined Score. (*[*https://maayanlab.cloud/Enrichr/enrich?dataset=3aa1862e2863900638215d376cc53841*](https://maayanlab.cloud/Enrichr/enrich?dataset=3aa1862e2863900638215d376cc53841)*)*

**Figure S14**


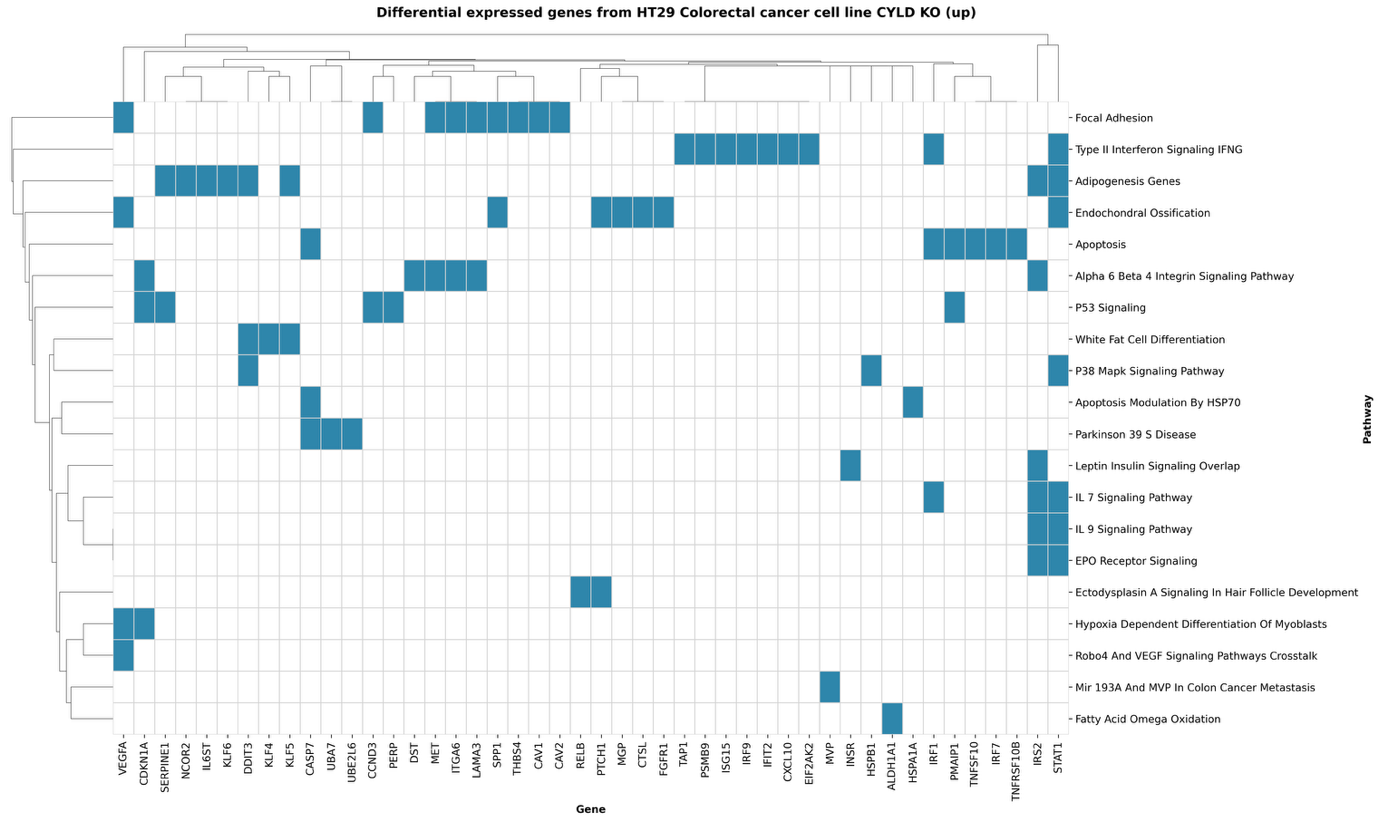


***Figure S14.*** ***Heatmap with hierarchical clustering depicting Enrichr pathway enrichment on differentially expressed genes (up) from the CYLD Knock-Out (KO) experiment on the HT29 Colorectal cancer cell line, as retrieved from the L2S2 web-tool.*** *The heatmaps show the top 20 pathways, thresholded by the Combined Score. (*[*https://maayanlab.cloud/Enrichr/enrich?dataset=f4110547ced85180725d0ef2ab863ae0*](https://maayanlab.cloud/Enrichr/enrich?dataset=f4110547ced85180725d0ef2ab863ae0)*)*

**Figure S15**


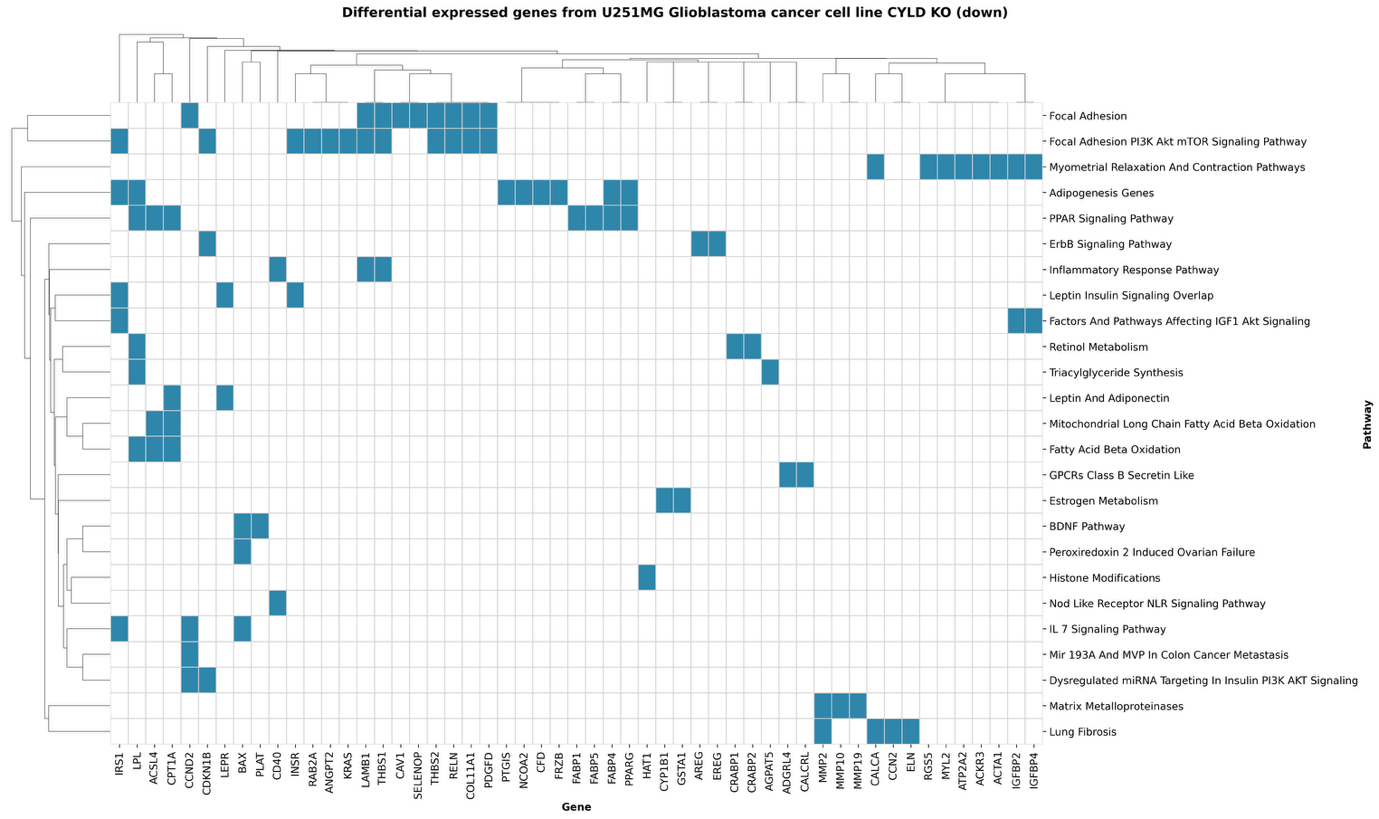


***Figure S15.*** ***Heatmap with hierarchical clustering depicting Enrichr pathway enrichment on differentially expressed genes (down) from the CYLD Knock-Out (KO) experiment on the U251MG Glioblastoma cancer cell line, as retrieved from the L2S2 web-tool.*** *The heatmaps show the top 25 pathways, thresholded by the Combined Score. (*[*https://maayanlab.cloud/Enrichr/enrich?dataset=24dc72acfff8dada22e12efb8b13afaa*](https://maayanlab.cloud/Enrichr/enrich?dataset=24dc72acfff8dada22e12efb8b13afaa)*)*

**Figure S16**

***
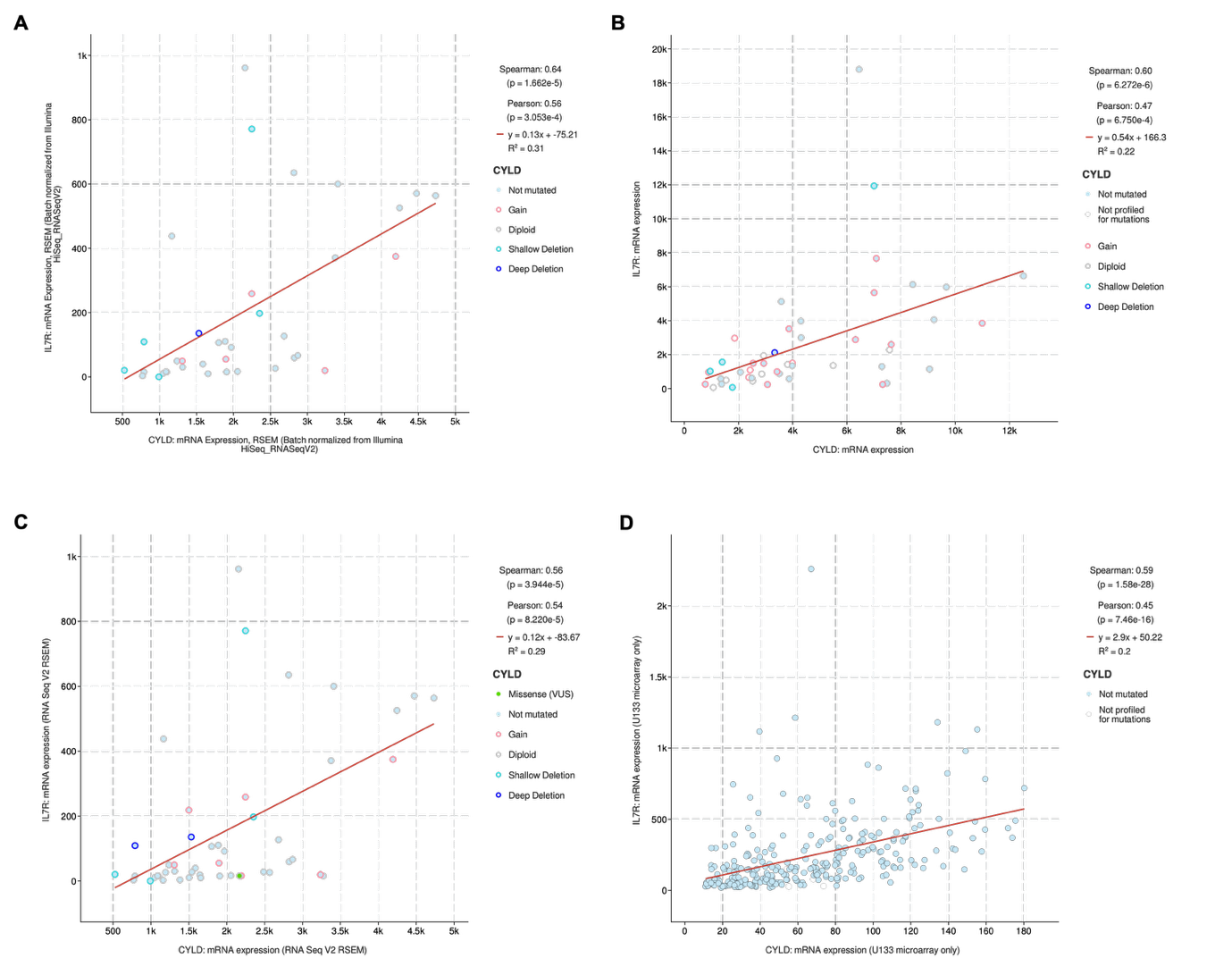
***

***Figure S16.*** ***Correlation of CYLD expression with IL-7 pathway.*** *CYLD and IL7R mRNA expression levels show a significant linear correlation in four different datasets of lymphoid diseases in cBioPortal public database (****A:*** *Diffuse Large B-Cell Lymphoma, TCGA, PanCancer Atlas;* ***B:*** *Diffuse Large B-Cell Lymphoma, NOS TCGA, GDC;* ***C:*** *Lymphoid Neoplasm Diffuse Large B-cell Lymphoma, TCGA, Firehose Legacy;* ***D:*** *Mature B-cell malignancies, MD Anderson Cancer Center).*

**Figure S17**

***Figure S17. Full and uncropped western blot for figure S1D.***

**Table S1. Antibodies & other reagents used for flow cytometry**

**Bone Marrow**

| **Antibody** | **Fluorophore** | **Clone** | **Dilution** | **Vendor** |
| --- | --- | --- | --- | --- |
| CD19 | APC-Cy7 | 6D5 | 1:100 | Biolegend |
| B220 | FITC | RA3-6B | 1:100 | Biolegend |
| c-kit (CD117) | PE | ACK45 | 1:100 | Biolegend |
| CD25 | BV421 | PC61 | 1:100 | Biolegend |
| IgM | Biotinylated | RMM-1 | 1:200 | Biolegend |
| CD3 | Pacific Blue | 500A2 | 1:50 | BD |
| CD4 | FITC | RM4-5 | 1:100 | Biolegend |
| CD8 | PE | 53-6.7 | 1:100 | Biolegend |
| IL-7Rα | APC | A7R34 | 1:50 | Biolegend |
| Streptavidin | PE |  | 1:200 | Biolegend |

**Spleen**

| **Antibody** | **Fluorophore** | **Clone** | **Dilution** | **Vendor** |
| --- | --- | --- | --- | --- |
| CD19 | APC-Cy7 | 6D5 | 1:100 | Biolegend |
| B220 | BV510 | RA3-6B | 1:100 | Biolegend |
| IgD | FITC | 11-26c.2a | 1:100 | Biolegend |
| CD21 | BV421 | 7E9 | 1:100 | Biolegend |
| CD23 | PE | B3B4 | 1:100 | Biolegend |
| IgM | Biotinylated | RMM-1 | 1:200 | Biolegend |
| Streptavidin | APC |  | 1:200 | Biolegend |
| CD3 | Pacific Blue | 500A2 | 1:50 | BD |
| CD4 | FITC | RM4-5 | 1:100 | Biolegend |
| CD8 | PE | 53-6.7 | 1:100 | Biolegend |

**Table S2. Cellular subpopulations identified by specific surface marker expression**

**Bone Marrow**

| **Population** | **Surface markers** |
| --- | --- |
| B cells | CD19^+^B220^+^ |
| Pro-B cells | CD19^+^B220^+^c-kit^+^CD25^-^ |
| Pre-B cells | CD19^+^B220^+^c-kit^-^CD25^+^ |
| Immature B cells | CD19^+^B220^low^IgM^int^ |
| Mature B cells | CD19^+^B220^high^IgM^int^ |
| Transitional B cells | CD19^+^B220^int^IgM^high^ |
| T cells | CD3^+^D19^-^ |
| T helper cells | CD3^+^D4^+^CD8^-^ |
| Cytotoxic T cells | CD3^+^D4^-^CD8^+^ |

**Spleen**

| **Population** | **Surface markers** |
| --- | --- |
| B cells | CD19^+^B220^+^ |
| T1 B cells | B220^+^IgM^high^IgD^low^ |
| T2 B cells | B220^+^IgM^int^IgD^int^ |
| Mature B cells | B220^+^IgM^low^IgD^high^ |
| Marginal zone progenitor B cells | CD19^+^CD23^int^CD21^int^ |
| Marginal zone B cells | CD19^+^CD23^-/low^CD21^high^ |
| Follicular zone B cells | CD19^+^CD23^high^CD21^low^ |
| T cells | CD3^+^D19^-^ |
| T helper cells | CD3^+^D4^+^CD8^-^ |
| Cytotoxic T cells | CD3^+^D4^-^CD8^+^ |

**Table S3. Antibodies used for immunoblotting**

| **Antibody** | **Dilution** | **Catalog No.** | **Vendor** |
| --- | --- | --- | --- |
| rabbit anti-CYLD | 1:500 | SAB4200061 | Sigma-Aldrich |
| mouse anti-β-Actin | 1:40000 | A3854 | Sigma-Aldrich |
| mouse anti-rabbit IgG-HRP | 1:1000 | sc-2357 | Santa Cruz Biotech |

**Table S4. Primer sequences used for qPCR**

| **Gene** | **Forward (5’-3’)** | **Reverse (5’-3’)** |
| --- | --- | --- |
| Cyld^2-3^ | CCTCAAATCCGATAGAGCATGG | AATGAACTCGCTGTAAGTCCC |
| Cyld^11-12^ | GGAAGGTTTAGAGATAATGATTGGAAAG | TCCAGGGCAGAACTAAAAGC |
| Il7ra | GGATGGAGACCTAGAAGATG | GAGTTAGGCATTTCACTCGT |
| Mcl1 | AAGAGGCTGGGATGGGTTTGT | AGTCCCCTATTGCACTCACAAG |
| Il7 | GGAATTCCTCCACTGATCCTTG | CTCAGTAGTCTCTTTAGG |
| Icam1 | CAATTTCTCATGCCGCACAG | AGCTGGAAGATCGAAAGTCCG |
| Rag1 | GCTATCTCTGTGGCATCGAGTG | GGTGTTGAATTTCATCGGGTG |
| Rag2 | GGCCTTCAGTGCCAAAATAA | TGTTACCATCTGCAGGGACA |
| Foxo1 | ACATTTCGTCCTCGAACCAGCTCA | ATTTCAGACAGACTGGGCAGCGTA |
| Ikzf1 | CCACAACGAGATGGCAGAAGAC | GGCATGTCTGACAGGCACTTGT |
| Pax5 | AGAGTATTCAGCCATGGCTTCA | GTGCAGAGTAGCTGCCCTGT |
| Ikzf3 | CCGAGATGGGAAGTGAGAGA | CGCTTCTCACCGATGAATTT |
| Ebf1 | GCCTTCTAACCTGCGGAAATCCAA | GGAGCTGGAGCCGGTAGTGGAT |
| Tcfe2a | CCAGTCTCAGAGAATGGCAC | CCTTCGCTGTATGTCCGGCTAG |
| Spif1 | CCCGGATGTGCTTCCCTTAT | TCCAAGCCATCAGCTTCTCC |
| Flt3 | TGGGACACCATGACAACATC | GGAATTTGAATGTGCCTGGA |
| Runx1 | CTCGGCAGAACTGAGAAATG | GGTGATGGTCAGAGTGA |
| Gfi1 | AGCGTCGGAGAAGTCACTGT | CAGGTCAGACCCAGCAAGAC |
| Irf4 | GTGGAAACACGCGGGCAAGC | GGCTCCTCTCGACCAATTCCTCA |
| Irf8 | AGAGGGAGACAAAGCTGAACCAGCC | CCACGCCCAGCTTGCATTTT |
| Miz-1 | AGGCACACTGTCTGAGAAGAGA | TGGTTCAGCTGCTCCAAGA |
| Rn18s | GTAACCCGTTGAACCCCATT | CCATCCAATCGGTAGTAGCG |

**Table S5.** A list of genes similar to CYLD was produced using mRNA Co-Expression from the ARCHS4 similarity matrix with Geneshot. The analysis can be retrieved by following this link: <https://playbook-workflow-builder.cloud/graph/0ae9d21b-46eb-9d32-acfa-37cbb81333f3/node/d0507d44-1385-438f-05cd-342d015f7d4f/extend>

| **Gene** | **ZScore** |
| --- | --- |
| **CREBRF** | 0.7272448539733887 |
| **TAGAP** | 0.7231678366661072 |
| **GVINP1** | 0.7141240239143372 |
| **ANKRD44** | 0.7129164338111877 |
| **PTPRC** | 0.7110047936439514 |
| **EVI2A** | 0.7101869583129883 |
| **CYTIP** | 0.7041268944740295 |
| **TRAF3IP3** | 0.697003185749054 |
| **GPR183** | 0.6969333291053772 |
| **HECA** | 0.6951957941055298 |
| **ITK** | 0.6933243870735168 |
| **P2RY10** | 0.6920024156570435 |
| **GBP5** | 0.6887673139572144 |
| **EVI2B** | 0.6865221261978149 |
| **TLR1** | 0.6824578642845154 |
| **TRIM22** | 0.6821753978729248 |
| **TNFSF8** | 0.6798892617225647 |
| **PYHIN1** | 0.6794345378875732 |
| **GPR65** | 0.6784263849258423 |
| **SP140** | 0.6778508424758911 |
| **SNX20** | 0.6763694882392883 |
| **C5orf56** | 0.6760272979736328 |
| **CLEC2D** | 0.6756357550621033 |
| **STAT4** | 0.6724189519882202 |
| **CD48** | 0.6692032217979431 |
| **GPR171** | 0.6674534678459167 |
| **TRAT1** | 0.6670221090316772 |
| **SAMD9L** | 0.6669551730155945 |
| **PTPN22** | 0.6668760180473328 |
| **GPR174** | 0.6661169528961182 |
| **LYST** | 0.665266752243042 |
| **SLAMF1** | 0.6639612317085266 |
| **IL16** | 0.6634511947631836 |
| **PATL2** | 0.6626979112625122 |
| **BTLA** | 0.6624481081962585 |
| **GBP4** | 0.6612327098846436 |
| **ICOS** | 0.6609808802604675 |
| **IL10RA** | 0.6606728434562683 |
| **GIMAP5** | 0.6606512665748596 |
| **SLAMF6** | 0.659713089466095 |
| **SNRK** | 0.6593839526176453 |
| **CTSS** | 0.6587648391723633 |
| **IL7R** | 0.6580154299736023 |
| **SLFN12L** | 0.6578955054283142 |
| **KCNA3** | 0.656657874584198 |
| **CD226** | 0.6562564969062805 |
| **THEMIS** | 0.6561174392700195 |
| **ARHGAP15** | 0.6560085415840149 |
| **PIK3IP1** | 0.6558744311332703 |
| **GIMAP7** | 0.6557879447937012 |
| **ITSN2** | 0.6557224988937378 |
| **SELL** | 0.6556026935577393 |
| **GIMAP2** | 0.654154896736145 |
| **FAM65B** | 0.653860867023468 |
| **CD6** | 0.6536708474159241 |
| **SASH3** | 0.6535254716873169 |
| **IPCEF1** | 0.6534613966941833 |
| **NLRC3** | 0.6524954438209534 |
| **GIMAP4** | 0.6499540209770203 |
| **SLA** | 0.6495130062103271 |
| **CASP1** | 0.6493923664093018 |
| **NLRC5** | 0.6487494707107544 |
| **CYTH4** | 0.6486613750457764 |
| **CD28** | 0.6482085585594177 |
| **SELPLG** | 0.6474418640136719 |
| **ZNF831** | 0.6474242806434631 |
| **TRAC** | 0.6455315947532654 |
| **CD2** | 0.6453742384910583 |
| **RP11-1094M14.8** | 0.644471287727356 |
| **STK17B** | 0.6442831754684448 |
| **CD53** | 0.6438205242156982 |
| **TMEM71** | 0.6431737542152405 |
| **CYTH1** | 0.6430011987686157 |
| **BIN2** | 0.6429947018623352 |
| **CD69** | 0.6426765322685242 |
| **LY9** | 0.6421709656715393 |
| **DOCK10** | 0.6415424346923828 |
| **BTN3A1** | 0.6409026384353638 |
| **JAML** | 0.6400389671325684 |
| **RASSF5** | 0.6389692425727844 |
| **N4BP2L1** | 0.638760507106781 |
| **LCP2** | 0.638759970664978 |
| **LRMP** | 0.638576328754425 |
| **ITGAL** | 0.6383735537528992 |
| **GIMAP6** | 0.637795090675354 |
| **RP11-686D22.10** | 0.6374328136444092 |
| **PLEK** | 0.637365996837616 |
| **2,00 VNN** | 0.6371232271194458 |
| **GIMAP8** | 0.6371160745620728 |
| **HCLS1** | 0.6354292631149292 |
| **CD5** | 0.6354078054428101 |
| **TOMM20P2** | 0.6353132128715515 |
| **CNOT6L** | 0.6342388987541199 |
| **LSP1** | 0.6340176463127136 |
| **CFLAR** | 0.6339592337608337 |
| **B2M** | 0.6337752938270569 |
| **GPR18** | 0.6336042881011963 |
| **IL12RB1** | 0.6335159540176392 |
| **GIMAP1** | 0.6334368586540222 |
| **CD84** | 0.633313000202179 |
| **CD40LG** | 0.6331549286842346 |
| **APBB1IP** | 0.6320134401321411 |
| **SAMSN1** | 0.631905198097229 |
| **APOL3** | 0.6313881278038025 |
| **ARHGAP30** | 0.6312735080718994 |
| **FYB** | 0.6305389404296875 |
| **HACD4** | 0.6295813918113708 |
| **TBX21** | 0.6294516921043396 |
| **TXK** | 0.629254162311554 |
| **GBP2** | 0.6291255354881287 |
| **CLEC2B** | 0.6289670467376709 |
| **CCR7** | 0.6282973289489746 |
| **PARVG** | 0.6279601454734802 |
| **BIRC3** | 0.6279247999191284 |
| **NCKAP1L** | 0.6275810599327087 |
| **ZBP1** | 0.6275120377540588 |
| **IL2RA** | 0.6275030374526978 |
| **FLI1** | 0.6269662976264954 |
| **CCL4** | 0.6267895102500916 |
| **XAF1** | 0.6262201070785522 |
| **FGL2** | 0.6258484721183777 |
| **LPXN** | 0.6257902383804321 |
| **ALOX5AP** | 0.6256151795387268 |
| **JAK2** | 0.6255389451980591 |
| **PSTPIP1** | 0.6254770755767822 |
| **IRF1** | 0.6254327297210693 |
| **ARHGAP9** | 0.6249430775642395 |
| **PIK3CG** | 0.6248978972434998 |
| **SRGN** | 0.6242161989212036 |
| **ARRDC5** | 0.6241295337677002 |
| **USP15** | 0.624116837978363 |
| **CDC42SE2** | 0.6229934096336365 |
| **DOCK2** | 0.6222933530807495 |
| **C16orf54** | 0.6220911145210266 |
| **TIGIT** | 0.6220342516899109 |
| **APOBEC3G** | 0.6219980716705322 |
| **AOAH** | 0.6215423941612244 |
| **E2F3P1** | 0.6207887530326843 |
| **TRAF1** | 0.6207727193832397 |
| **APOL6** | 0.6206467151641846 |
| **IKZF3** | 0.6205027103424072 |
| **CD244** | 0.6201905012130737 |
| **PARP8** | 0.6200370788574219 |
| **ARHGAP25** | 0.6199836730957031 |
| **SCML4** | 0.6194373965263367 |
| **LAPTM5** | 0.6190762519836426 |
| **TCP11L2** | 0.618193507194519 |
| **CTD-2370N5.3** | 0.6179787516593933 |
| **DENND4A** | 0.6177574992179871 |
| **ZC3H12D** | 0.6175343990325928 |
| **HLA-B** | 0.617342472076416 |
| **SLC12A6** | 0.6171750426292419 |
| **RASAL3** | 0.6169975399971008 |
| **RP11-693N9.2** | 0.6163281798362732 |
| **BCL2A1** | 0.6161344647407532 |
| **PAG1** | 0.6159356236457825 |
| **FGR** | 0.6156939268112183 |
| **CTLA4** | 0.6156410574913025 |
| **CCR5** | 0.6156395673751831 |
| **CCR4** | 0.6154853105545044 |
| **IL18RAP** | 0.615121066570282 |
| **CD96** | 0.6148574948310852 |
| **MYO1G** | 0.6147182583808899 |
| **GNLY** | 0.614364743232727 |
| **SH2D1A** | 0.61419677734375 |
| **CXorf21** | 0.6139400601387024 |
| **PARP15** | 0.6138453483581543 |
| **BTN3A3** | 0.6135697364807129 |
| **XRN1** | 0.6129758954048157 |
| **GPR132** | 0.6120879054069519 |
| **RCSD1** | 0.612064778804779 |
| **MEI1** | 0.6118501424789429 |
| **GMFG** | 0.6095750331878662 |
| **IKZF1** | 0.6092333197593689 |
| **FCRL3** | 0.6088849306106567 |
| **HAVCR2** | 0.6087628602981567 |
| **HLA-F** | 0.6082671284675598 |
| **CD3E** | 0.6080905199050903 |
| **TRBC1** | 0.608059823513031 |
| **S1PR4** | 0.6072146892547607 |
| **OAS2** | 0.6062200665473938 |
| **SLC9A9** | 0.6059901714324951 |
| **DOCK8** | 0.6052582859992981 |
| **CYSLTR2** | 0.6050165891647339 |
| **RASGRP1** | 0.6048535704612732 |
| **HSF5** | 0.6043709516525269 |
| **HLA-E** | 0.604164183139801 |
| **CRTAM** | 0.6033359169960022 |
| **IL21R** | 0.60326087474823 |
| **LAX1** | 0.6032083630561829 |
| **PHF11** | 0.6030917167663574 |
| **MX2** | 0.6026812195777893 |
| **GZMK** | 0.602584719657898 |
| **TESPA1** | 0.6025168299674988 |
| **IL2RG** | 0.6023836135864258 |
| **CD247** | 0.6019827127456665 |
| **KLRB1** | 0.6018201112747192 |
| **TNFRSF1B** | 0.6013489961624146 |
| **CD300A** | 0.6006031632423401 |
| **LY75** | 0.6002746224403381 |
